# Abrupt and Reversible Stretching in an Azobenzene Single Crystal via Thermal Phase Transition

**DOI:** 10.1002/advs.202512603

**Published:** 2025-10-14

**Authors:** Minghao Gao, Dennis Kwaria, Emi Uchida, Hiroyuki Minamikawa, Rie Haruki, Reiji Kumai, Youfeng Yue, Yasuo Norikane

**Affiliations:** ^1^ Core Electronics Technology Research Institute National Institute of Advanced Industrial Science and Technology (AIST) 1‐1‐1 Higashi Tsukuba Ibaraki 305–8565 Japan; ^2^ Graduate School of Pure and Applied Sciences University of Tsukuba 1‐1‐1 Tennodai Tsukuba Ibaraki 305–8571 Japan; ^3^ Research Institute for Chemical Process Technology National Institute of Advanced Industrial Science and Technology (AIST) 1‐1‐1 Higashi Tsukuba Ibaraki 305–8565 Japan; ^4^ Photon Factory Institute of Materials Structure Science (IMSS) High Energy Accelerator Research Organization (KEK) 1‐1 Oho Tsukuba Ibaraki 305–0801 Japan

**Keywords:** anisotropic expansion, CH–π interactions, photothermal effect, single‐component organic crystals, thermal single crystal‐single crystal phase transitions

## Abstract

Mechanically responsive crystals are promising for actuators and microrobotics; however, achieving large reversible deformation with high durability remains challenging. Herein, A single‐component azobenzene crystal is reported to exhibit an abrupt and reversible stretching of over 8% along its long axis, driven by a thermally induced single‐crystal‐to‐single‐crystal phase transition. Single‐crystal X‐ray diffraction revealed a significant change in molecular packing distance along the long axis accompanied by the reorientation of CH–π interactions, leading to large macroscopic stretching/shrinking. Furthermore, visible light (435 nm) induced rapid, localized, and reversible stretching of the crystal via the photothermal effect, enabling the remote control of microparticle motion. This study reveals a rare combination of large anisotropic deformation, reversibility, and light responsiveness in a single‐component organic crystal, offering a new molecular platform for advancing micro‐energy conversion and soft robotics.

## Graphical abstract

Crystals of azobenzene compounds exhibit abrupt and reversible stretching and shrinking in response to thermal and photo stimulation. The single‐crystal‐to‐single‐crystal phase transition between two polymorphs involving reorientation of CH–π hydrogen interactions are demonstrated. Furthermore, the abrupt photoinduced stretching of the crystals is used to trigger microparticle transportation.

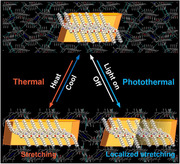

## 1 Introduction

Mechanically responsive materials have been extensively studied as dynamic systems capable of undergoing significant shape transformations and locomotion in response to external stimuli, making them attractive for applications in intelligent and adaptive devices.^[^ ^1^, ^2^, ^3^, ^4^, ^5^, ^6^, ^7^, ^8^ ^]^ Among these, mechanically responsive molecular crystals have emerged as promising candidates for actuators, microrobotics, and sensors owing to their well‐ordered structures and cooperative molecular behaviors.^[^ ^4^, ^5^, ^6^, ^9^, ^10^, ^11^ ^]^ Their mechanical response is closely tied to their molecular packing and intermolecular interactions.^[^ ^2^, ^3^, ^4^, ^5^, ^6^ ^]^ In particular, noncovalent interactions enable significant mechanical deformation through cooperative molecular rearrangement. Moreover, the dense and anisotropic packing of crystals allows for the efficient amplification of molecular‐scale changes into macroscopic mechanical motions, offering high energy‐conversion efficiency and rapid actuation in response to external stimuli.^[^ ^5^ ^]^

A widely explored strategy to realize reversible and rapid mechanical deformation involves exploiting crystalline phase transitions, which are typically triggered by light or heat.^[^ ^4^, ^5^, ^6^ ^]^ These transitions can be driven by the collective motion of molecular motors in complexes or co‐crystals, or by reversible chemical transformations such as isomerization or cycloaddition within the crystal lattice.^[^ ^12^, ^13^, ^14^, ^15^, ^16^, ^17^, ^18^, ^19^, ^20^, ^21^, ^22^, ^23^, ^24^, ^25^ ^]^ In addition, polymorphic transitions offer an effective means of achieving rapid mechanical deformations in molecular crystals via changes in molecular conformation and packing structure.^[^ ^26^, ^27^, ^28^, ^29^, ^30^, ^31^, ^32^ ^]^ However, such transitions often induce acute lattice distortion and mechanical stress, leading to considerable cracks or irreversible damage in the crystals (e.g., the salient effect), which compromises the durability and reversibility of the mechanical motions.^[^ ^6^, ^10^, ^33^, ^34^, ^35^, ^36^ ^]^ Therefore, achieving both rapid and large macroscopic shape changes while maintaining high fatigue resistance remains a significant challenge in molecular crystals.^[^ ^4^, ^5^, ^6^, ^12^, ^13^, ^37^, ^38^ ^]^

Herein, we report the remarkably large, rapid, and reversible anisotropic expansion (stretching) of a single‐component azobenzene crystal upon thermal stimulation. The stretching along the long crystal axis of over 8% is based on a thermally induced single‐crystal‐to‐single‐crystal (SCSC) phase transition between polymorphs with subtle molecular conformational changes, leading to an extraordinary rearrangement of the crystal packing structure. The SCSC phase transition is fully reversible with thermal hysteresis, leading to abrupt stretching/shrinking of the crystal. Furthermore, we employed a single on–off exposure to visible light (435 nm) to remotely and reversibly trigger the stretching/shrinking of the crystal via a localized photothermal effect. Below the SCSC phase transition temperature, the crystal abruptly stretched under blue light irradiation and rapidly shrank to its original state upon light cessation. This rapid back‐and‐forth motion exhibited no hysteresis and could be repeated over hundreds of cycles without significant damage. This highly anisotropic stretching/shrinking motion was demonstrated to trigger the directional mechanical movement of microparticles. We anticipate that the photoinduced motion observed in these crystals under heat or light stimuli can be applied to the remote operation of durable microrobots.

## 2 Results and Discussion

### 2.1 Thermal Induced Anisotropic Crystal Expansion and Contraction

**Figure 1:**
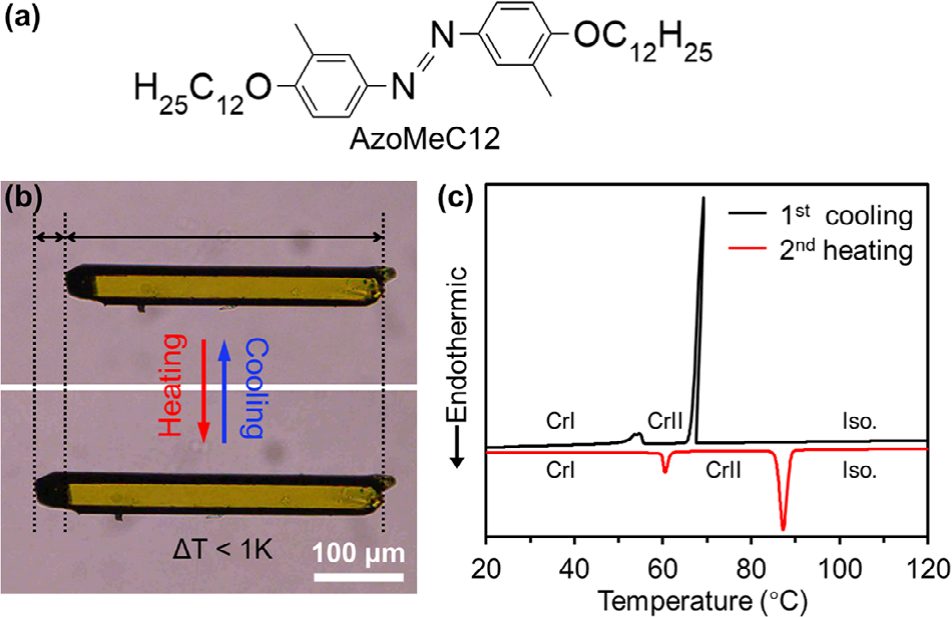
a) Chemical structure of compound AzoMeC12. b) Thermal stretching of a AzoMeC12 crystal between CrI and CrII. c) Thermogram of AzoMeC12 upon the first cooling and second heating scanning at 5 °C min^−1^. CrI, crystal phase I; CrII, crystal phase II; Iso, isotropic liquid phase.

Crystals of 3,3′‐dimethyl‐4,4′‐didodecyloxy azobenzene (AzoMeC12) exhibit a symmetric configuration with long alkoxy chains and methyl groups at para‐ and meta‐ positions of the azobenzene chromophore (**Figure** **1**a). AzoMeC12 was synthesized following a previously reported procedure,^[^ ^39^ ^]^ and long prismatic single crystals were obtained by recrystallization (Figure 1b). Upon heating to ≈60°C, the crystal exhibits an abrupt anisotropic expansion (stretching) of over 8% along its long axis within a 1 K interval, followed by a reverse contraction (shrink) upon cooling (Figure 1b). Differential scanning calorimetry (DSC) was performed to characterize the thermal properties of AzoMeC12. As shown in Figure 1c, the DSC thermograms revealed a sharp exothermic peak at 69°C and an endothermic peak at 87°C during the first cooling and second heating cycles, respectively, which correspond to the freezing and melting processes of the compounds. In addition to these sharp peaks, smaller endothermic and exothermic peaks appear at ≈60 and 54°C, respectively. These sharp peaks and a thermal hysteresis loop at ≈5°C indicate that a first‐order phase transition occurred (Figure S1, Supporting Information). When the temperature exceeded the phase transition point, microscopic observations revealed an abrupt and smooth propagation of stretching along the length of the crystal. During heating and cooling, a distinct boundary was formed between the elongated and non‐elongated crystalline regions, extending from one end of the crystal to the other (Movie S1, Supporting Information). These observations clearly indicate that the change in crystal shape can be attributed to a phase transition. Therefore, these weaker peaks correspond to the reversible SCSC phase transitions between low temperature crystal phase I (CrI) and high‐temperature crystal phase II (CrII), which drive the macroscopic stretching and shrinking of the crystals (Figure 1c).

**Figure 2:**
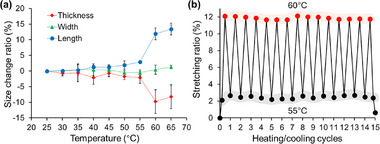
a) Temperature‐dependent size change of the crystals in different directions compared with their size at 25°C. b) Stretching ratio of crystal along long axis at 55 and 60°C relative to that at 25°C during repeated heating/cooling cycles. The crystal was eventually cooled to 25°C.

We measured the changes in crystal size along the thickness, length, and width as the temperature increased from 25 to 65°C using a laser scanning microscope (Figure S2, Supporting Information). As shown in **Figure** **2**a, the crystal length gradually increased by ≈3% at 55°C, followed by a sharp increase of 12% at 65°C. Simultaneously, the crystal height decreased significantly (by 8%), whereas the width increased slightly (by 1%). This elongation ratio is relatively high among single component molecular organic crystals exhibiting thermally induced stretching through phase transition (Figure S3, Supporting Information).^[^ ^12^, ^13^, ^27^, ^28^, ^29^, ^30^, ^31^ ^]^ The crystal size and stretching ratio were also measured by repeatedly heating a single crystal of AzoMeC12 to 60°C and cooling it to 55°C (Figure 2b). After 10 heating/cooling cycles, the stretching ratio remained above 12%, and the reverse process exhibited full recovery of the original crystal size, demonstrating a high degree of reversibility and repeatability of stretching. This complete switching, owing to the SCSC phase transition, was consistent with the DSC measurements under cyclic heating and cooling (Figure S1, Supporting Information). The response time, defined as the period between the onset and completion of the stretching/shrinking process, was measured for a single crystal at varying heating/cooling rates (Figure S4, Supporting Information). Upon repeated thermal cycling, both the stretching and shrinkage responses were generally completed within 1 s. The response time exhibited no correlation with the heating/cooling rates.

### 2.2 Single‐Crystal Structures of CrI and CrII

**Figure 3:**
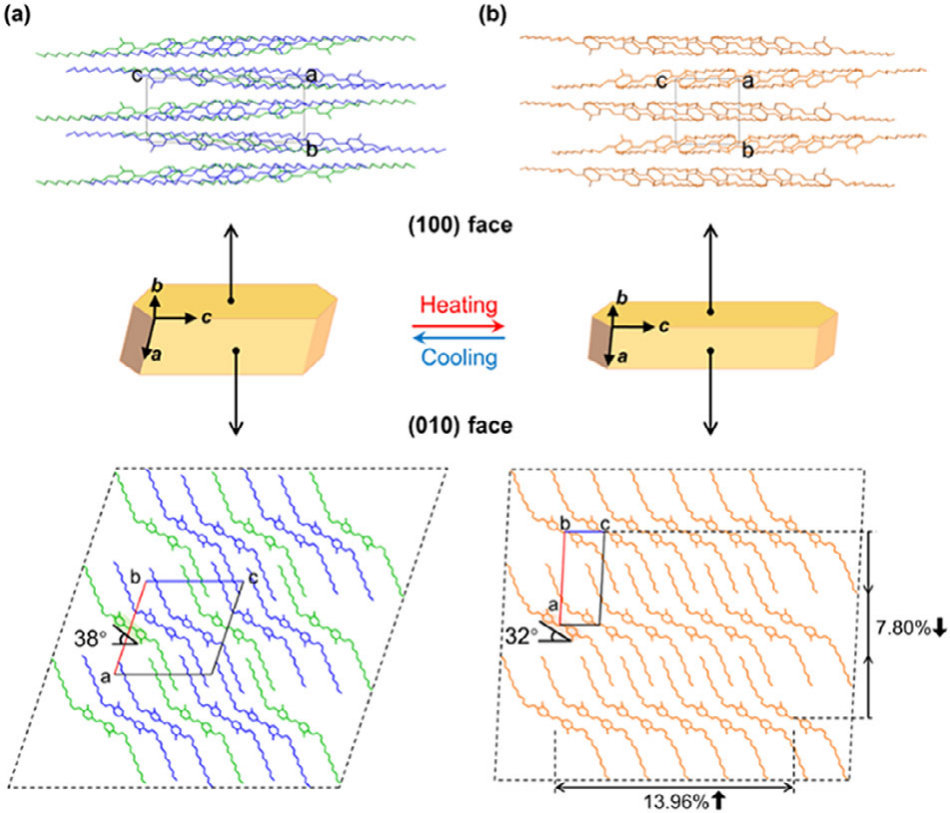
Single‐crystal structures of two phases and Illustration of crystal shapes and packing in AzoMeC12 crystal. From top to bottom: crystal structure viewed from the (100) face, crystal shape, and crystal structure viewed from the (010) plane in a) CrI and b) CrII phases, highlighting changes in packing distance and the tilt angle of azo moieties. Hydrogen atoms are omitted for clarity (CrIA conformer in blue, CrIB in green, CrIIC in orange).

To determine the crystal structures during the phase transition, single‐crystal X‐ray diffraction (SCXRD) analyses were conducted at 170, 298, and 338 K (Table S1, Supporting Information). The similar unit cell parameters at 170 K and room temperature (298 K) indicate that the crystal retains the same CrI phase within this temperature range. The crystal structure measured at 298 K was difficult to resolve fully owing to molecular disorder, whereas the structure was successfully determined at 170 K (**Figure** **3**a). The CrI phase consists of two distinct conformers: asymmetric conformer A (CrIA) and centrosymmetric conformer B (CrIB) (Figure 3a; Figure S5, Supporting Information). As evidenced by the dihedral angles of the azo moiety shown in Figure S5 (Supporting Information), CrIA exhibits a more strained geometry compared to CrIB. At 338 K, the unit cell volume decreases to approximately one‐third of that in the CrI phase, indicating a structural transition to the CrII phase (Table S1, Supporting Information). The CrII phase consists of a single conformer (CrIIC) (Figure 3b). Although relatively high disorder was observed, CrIIC exhibited a symmetric molecular structure, as shown in Figure S5 (Supporting Information). A common structural feature of the three conformers is that the C–O–C alkoxy chains bend away from the meta‐methyl groups on the benzene rings due to steric hindrance. The alkyl chains then twisted out of the plane of the azo core, forming a zigzag conformation (Figure S6, Supporting Information). The dihedral angles of the azo groups and alkoxy chains in the CrIIC molecule lie between those of the CrIA and CrIB molecules (Figure S5, Supporting Information). Therefore, upon heating, the molecular conformation changed to a more relaxed state. Similar conformational relaxation upon heating was observed in an azobenzene crystal exhibiting a photosensitive effect.^[^ ^16^ ^]^

SCXRD revealed that the crystallographic *c*‐axis correlated with the long axis of the crystal, as the (100) and (010) faces corresponded to the top‐ and side‐view planes of the crystal, respectively (Figure 3; Figure S7, Supporting Information). Thus, the stretching/shrinking behavior shown in Figure 1b is along the *c*‐axis. The molecular packing on the (100) face exhibited a lamellar structure along the *b*‐axis in both the CrI and CrII crystal phases. From the side view, the (010) face also shows a lamellar packing, with alternating layers composed of azobenzene moieties exhibiting π–π stacking (Figure S8a,b, Supporting Information) and interdigitated long alkoxy chains held together by van der Waals (vdW) interactions (Figure 3). The azo molecules aligned along the *c*‐axis adopt a herringbone packing arrangement (Figure S8c,d, Supporting Information). Upon heating to the phase transition temperature, the CrI phase transformed into the CrII phase, leading to a significant increase of 13.96% in the packing distance along the *c*‐axis and a decrease of 7.8% along the *a*‐axis (Figure 3b). The azobenzene moieties aligned along the *c*‐axis were inclined forward by ≈6°, increasing the intermolecular packing distance. These anisotropic shape changes corresponded to the macroscopic crystal size changes measured using a laser scanning microscope (Figure S2 and Table S2, Supporting Information).

**Figure 4:**
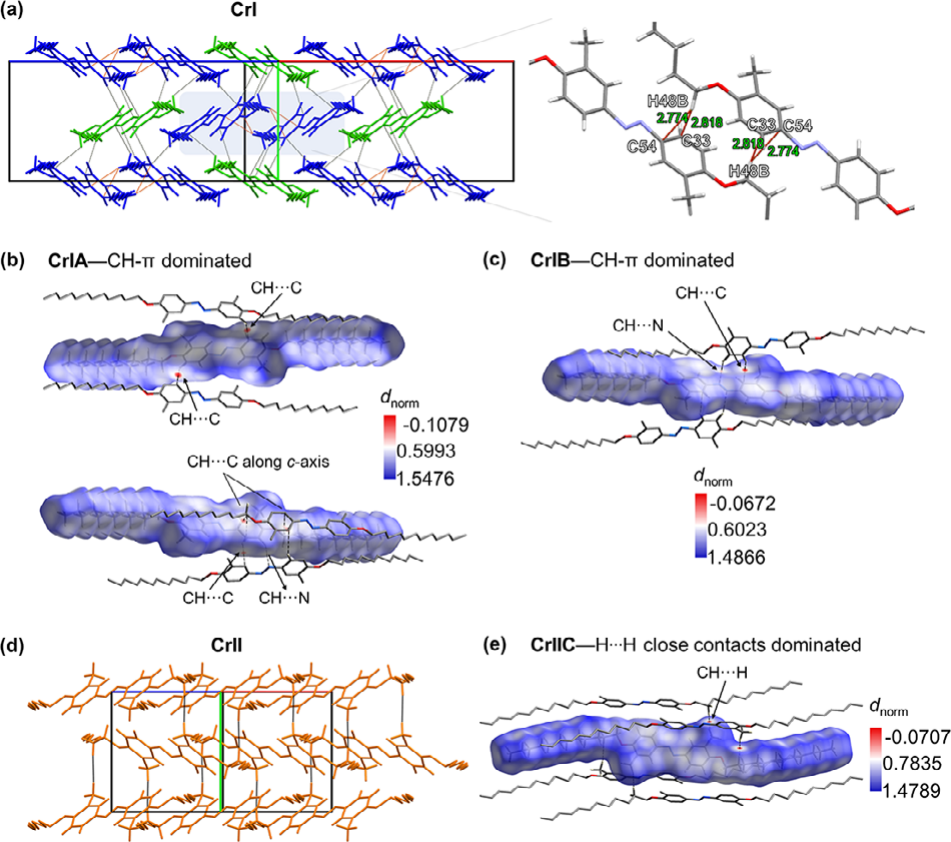
a) Crystal packing showing short contact interactions in CrI (left, CrIA in blue and CrIB in green) and an enlarged region (right) of two CrIA molecules. Red dashed lines represent CH–π short contact interactions; the length unit is Å. HFS with *d_norm_* properties employed on b) CrIA and c) CrIB conformers. d) Crystal packing showing short contact interactions network in CrII phase. e) HFS with *d_norm_* properties employed on CrIIC conformer. The prominent interaction types on HFS are highlighted.

Intermolecular interactions play a key role in the rearrangement of the crystal structure during the SCSC phase transition. Hirshfeld surface (HFS) analysis was performed using CrystalExplorer 21 to clarify the dominant interactions in different conformers and phases.^[^ ^40^, ^41^ ^]^ The fingerprint plots of the Hirshfeld surface show that in all three conformers, the contribution proportion of H…H (> 80%) and C…H/H…C (>10%) interactions dominate the intermolecular interactions in AzoMeC12 (Figure S9, Supporting Information). H…H interactions correspond to vdW forces, while C…H/H…C interactions correspond to π–π interactions and CH–π weak hydrogen interactions. In addition to the aforementioned vdW interactions and π–π interactions, the difference in short contact (<sum of vdW radii) intermolecular interactions in two phases (visualized using Mercury software) was investigated (**Figure** **4**; Figure S10, Supporting Information). Notably, a network of CH–π short contact interactions among adjacent molecules are present in the CrI phase but absent in the CrII phase (Figure 4a, red dashed line). Mapping the normalized contact distance, (*d_norm_*), quantitative property derived from the HFS of each conformer, reveals red regions corresponding to the most dominant short contact interactions, while the white and blue colorations indicate less dominant short contact interactions (Figure 4b,c,e). The CrI phase contains CH–π short contact interactions (CH…C and CH…N contacts) as dominant interactions (Figure 4b,c). However, in the CrII phase, the short contacts are no longer CH–π interactions but H…H contacts (Figure 4e). A close inspection of the CrI phase reveals that a specific type of CH–π interaction (red dashed line, CH…C) between adjacent CrIA conformers (framed) forms along the *c*‐axis with relatively shorter distances (Figure 4a; Figure S10a, Supporting Information). Such interlocking‐type CH–π short contacts are not present in CrIB conformers. Consequently, the two CrIA conformers form a centrosymmetric molecular pair with a twisted conformation (Figure S5, Supporting Information). These relatively weak interactions serve as a soft medium for the crystal‐packing structure, allowing dynamic conformational freedom and the potential for drastic reorientation. The interlocked CrIA molecular pairs acted as entropy reservoir units. Heating overcame the energy barriers of these molecular pairs, leading to the reorientation of CH–π interactions. This reorientation triggered a phase transition and was amplified by a macroscopic anisotropic shape change.

### 2.3 Temperature‐Dependent Powder X‐ray Diffraction (PXRD)

**Figure 5:**
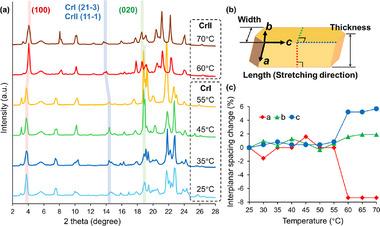
a) Temperature‐dependent PXRD patterns of crystals with representative diffraction peaks traced for corresponding crystal faces (100), (22‐3)/(12‐1), and (020). b) Correlation between macroscopic and microscopic crystal size. The colored dashed lines correspond to interplanar spacing directions. c) Interplanar spacing change of representative faces (100), (22‐3)/(12‐1), and (020) in CrI and CrII under varying temperatures.

To further investigate the crystal behavior during the SCSC phase transition, powder X‐ray diffraction (PXRD) was performed at varying temperatures, and the obtained PXRD patterns were compared with the simulated PXRD patterns derived from single‐crystal structural data (170 K for CrI and 338 K for CrII). **Figure** **5**a shows the PXRD patterns obtained at temperatures ranging from 25 to 70°C for the same sample. The distinct change in the diffraction patterns between 55 and 60°C indicates the occurrence of an SCSC phase transition. By comparing the measured PXRD patterns at 25 and 60°C with the simulated PXRD patterns, three representative peaks corresponding to the crystal faces (100), (21‐3)/(11‐1), and (020) were selected to identify changes in packing distance during the phase transition (Figure S11, Supporting Information). These three peaks are represented by different colors in Figure 5a, and their corresponding interplanar spacings were calculated using Bragg's equation. The interplanar spacings, shown in red, blue, and green in Figure 5a, correspond to the dimensional changes in the crystal thickness, length, and width, respectively (Figure S12, Supporting Information). The [21‐3]/[11‐1] directions are not perpendicular to the (001) face; therefore, they do not directly indicate that the packing distance changes along the *c*‐axis. However, they represent the spacing between adjacent alkoxy chains in the packing columns, indirectly reflecting the *c*‐axis packing distance (Figure 5b).

The changes in the interplanar spacing are plotted in Figure 5c. Between 55 and 60°C, the distinct changes in packing distances along the unit cell axes, calculated from the diffraction angle changes, correspond to the SCSC phase transition (Figure 5c). Along the *a*‐axis, the packing distance abruptly decreases, whereas along the *c*‐axis, it abruptly increases. Below the phase transition temperature, the tendency of the blue plots representing the packing distance along the *c*‐axis exhibits a consistent change with the measurements of the stretching ratio of the crystal. This is consistent with the change in crystal length measured using a laser scanning microscope (Table S2, Supporting Information).

### 2.4 Light‐Induced Abrupt Stretching/Shrinking of a Single Crystal and its Application in Microparticle Transport

**Figure 6:**
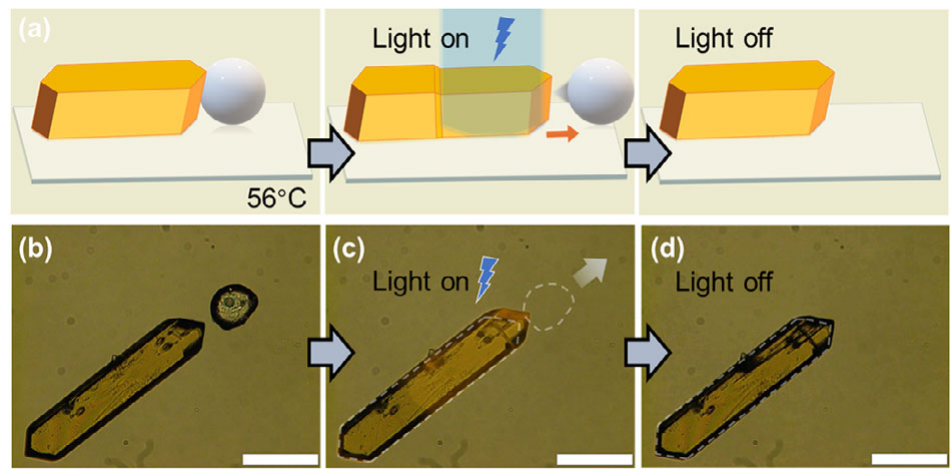
a) Illustration of the light‐induced crystal stretching used to trigger the movement of a silica gel microparticle. Photographs of AzoMeC12 crystal close to a silica gel microparticle with a diameter of ≈130 µm. b) with 435 nm light (3.6 W cm^‐^^2^) off and c) on and d) off at 56°C (the white dashed lines represent the positions of the crystal and silica gel microparticle before irradiation). The crystal is placed on an unmodified cover glass. Scale bars indicate 200 µm.

The rapid and reversible anisotropic expansion of these crystals induced by thermal stimuli can be applied to the mechanical output. Instead of using heat, the light‐induced process allows for noncontact manipulation with a high spatiotemporal resolution. In this study, we applied the photothermal effect to induce a phase transition (Figure S13, Supporting Information). As shown in **Figure** **6**a, by exposing the crystals to visible light (435 nm), reversible rapid stretching was successfully achieved without noticeable damage. The stretching/shrinking of the crystals can be triggered by turning on/off 435 nm light at temperatures slightly below the phase transition (55–56°C) (Figure S14 and Movie S2, Supporting Information). When the light was turned off, the crystal quickly recovered its shape. This on–off action of visible light induces reversible stretching and shrinking of the crystal without significant hysteresis, in contrast to thermal phase transitions, where hysteresis is typically present. This stretching could be repeated over 200 cycles without significant damage to the crystals (Movie S3, Supporting Information). To clarify the origin of the stretching, the UV– vis spectra of the samples in both the solution and crystalline states were measured. In solution, AzoMeC12 molecules photoisomerize from the trans to the cis state under UV light and revert to the trans state under blue light (Figure S15, Supporting Information). However, at 56 and 57°C, no changes in the absorption spectra of crystalline film samples were observed with or without irradiation (Figure S16, Supporting Information). Therefore, stretching can be attributed to the photothermal effect rather than photoisomerization. Irradiation experiments were then conducted on a single‐crystal sample at varying temperatures and blue light intensities (Figure S17, Supporting Information). At temperatures below 55°C and light intensities lower than 3.6 W cm^‐^^2^, no significant crystal stretching was observed. Furthermore, at the same light intensity, an increase in the temperature resulted in a higher stretching ratio. Given the strong temperature dependence of the light‐induced stretching, the mechanical behavior of AzoMeC12 crystals can be precisely controlled. At the highest light intensity and 58°C, the crystal exhibited the highest irreversible stretching ratio, with no shrinkage occurring after the light was turned off. This irreversible stretching indicates that the photothermal effect induces a complete phase transition of the crystal. By contrast, the previously observed reversible stretching without hysteresis was likely attributed to an incomplete phase transition.

Interestingly, the dynamic expansion of a prismatic crystal can generate sufficient force to propel the nearby microparticles (Figure 6a). As shown in Movie S4 (Supporting Information), once the blue light was turned on, the irradiated region of the crystal snapped forward (<0.1 s), striking the microparticles like a cue hitting a billiard ball (Figure 6b,c). When the light was turned off, the crystal immediately contracted. This visible light‐controlled local stretching and shrinking of crystals can be used to control the movement of microparticles or even small objects with high precision, enabling advanced microrobotic systems with enhanced precision and versatility.

## 3 Conclusion

In conclusion, abrupt and reversible stretching/shrinking behavior was observed in an organic single crystal, driven by thermally induced SCSC phase transitions between the CrI and CrII polymorphs. SCXRD revealed that two conformers, A and B, exist in the low‐temperature phase CrI, both of which transform into the uniform conformer C in the high‐temperature phase CrII. During the SCSC phase transition, the packing distance along the *c*‐axis increased significantly, accompanied by the reorientation of weak hydrogen‐bonding networks. This results in anisotropic elongation and contraction of the crystal, manifesting as abrupt and reversible stretching and shrinking. Notably, photothermally induced local stretching of the crystal was achieved by visible light irradiation at temperatures below the phase transition temperature. The crystal maintained excellent durability under rapid stretching, with no significant damage observed after hundreds of cycles. The force generated by stretching was demonstrated to induce the movement of the silica gel microparticles. We expect that azobenzene crystals will inspire the molecular design of mechanically responsive crystals and find applications in micro‐energy conversion, capable of converting thermal and photon energy into mechanical energy.

## Conflict of Interest

The authors declare no conflict of interest.

## Supporting information



Supporting Information

Supplemental Movie 1

Supplemental Movie 2

Supplemental Movie 3

Supplemental Movie 4

## Data Availability

The data that support the findings of this study are available from the corresponding author upon reasonable request. The crystallographic data for compound AzoMeC12 at Cr1 and CrII phases have been deposited at the CCDC under deposition number CCDC 2494140 (170K, CrI phase), CCDC 2494147 (338K, CrII phase) and can be obtained via www.ccdc.cam.ac.uk.
