# Supplementary material for: Abrupt and Reversible Stretching in an Azobenzene Single Crystal via Thermal Phase Transition
Source: Adv Sci (Weinh). 2025 Oct 14;12(48):e12603. doi: 10.1002/advs.202512603 (PMC12752656; doi:10.1002/advs.202512603)
Supplement: Supplementary file 1 — Supporting Information [file ADVS-12-e12603-s001.docx]

**Supplementary Information**

**Abrupt and Reversible Stretching in an Azobenzene Single Crystal via Thermal Phase Transition**

Minghao Gao, Dennis Kwaria, Emi Uchida, Hiroyuki Minamikawa, Rie Haruki, Reiji Kumai, Youfeng Yue*, Yasuo Norikane*

E-mail: y-norikane@aist.go.jp; yue-yf@aist.go.jp

**Table of Contents**

[**1.** **Materials** 2](#_Toc208606277)

[**2.** **Experimental methods** 2](#_Toc208606278)

[**Synthesis of 3,3’-dimethyl-4,4’-didodecyloxy azobenzene (1)** 2](#_Toc208606279)

[**Recrystallization** 3](#_Toc208606280)

[**Characterizations** 3](#_Toc208606281)

[**3D laser scanning microscopy** 3](#_Toc208606282)

[**Single-crystal X-ray diffraction (SXRD) analysis** 4](#_Toc208606283)

[**Temperature-dependent powder X-ray diffraction (PXRD) analysis** 4](#_Toc208606284)

[**Statistical analysis** 4](#_Toc208606285)

[**3.** **Other supporting figures and tables** 5](#_Toc208606286)

[**DSC data** 5](#_Toc208606287)

[**3D scanning of crystal size analysis** 6](#_Toc208606288)

[**Stretching ratio comparison** 7](#_Toc208606289)

[**Heating/cooling rate effect on crystal stretching/shrinking speed** 8](#_Toc208606290)

[**Crystal structures and intermolecular interactions** 9](#_Toc208606291)

[**Simulated and measured PXRD** 17](#_Toc208606292)

[**Photothermal effect** 19](#_Toc208606293)

[**Photothermal control of crystal stretching/shrinking** 20](#_Toc208606294)

[**UV–vis absorption spectra** 21](#_Toc208606295)

[**Solid-state absorption spectra** 22](#_Toc208606296)

[**Effects of light intensity and temperature on crystal stretching/shrinking** 23](#_Toc208606297)

[**4.** **References** 23](#_Toc208606298)

# **Materials**

4-Amino-*o*-cresol, *o*-cresol, 1-bromododecane, and 18-crown-6-ether were purchased from Tokyo Chemical Industry. Dehydrated acetonitrile, dehydrated dichloromethane, and dehydrated tetrahydrofuran were purchased from KANTO CHEMICAL Co., Inc. Sodium nitrite, dichloromethane, ethanol, acetic acid, sodium hydrogen carbonate, and anhydrous magnesium sulfate were purchased from Kishida Chemical Co., Ltd. Anhydrous potassium carbonate, and Celite No.503 were purchased from Wako Pure Chemical Corporation. All reagents were used as received. For column chromatography, Silica gel 60N (Kanto Chemical) was used. For NMR spectroscopy, chloroform-d (99.8 atom % D + 0.03 % (v/v) TMS, Merk KGaA) and dimethyl sulfoxide-d_6_ (99.9 atom % D + 0.03 % (v/v) TMS, Merk KGaA) were used. For absorption spectroscopy, chloroform (spectroscopic grade) was purchased from Dojindo Laboratories. Cover glass (Matsunami Glass Ind., Ltd. Square microscope cover glass No.1, 18 mm × 18 mm) was used for the glass substrate.

# **Experimental methods**

## **Synthesis of 3,3’-dimethyl-4,4’-didodecyloxy azobenzene (1)**

**1** was synthesized based on a previously reported method.^[1]^


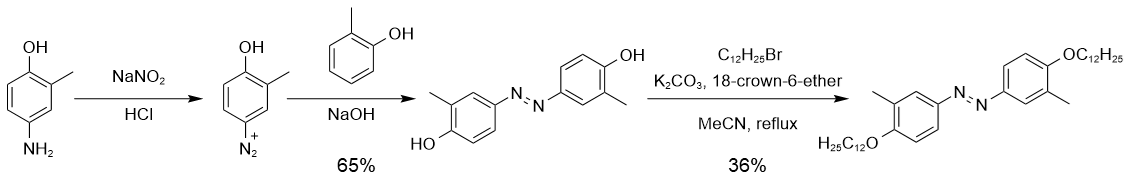


Synthesis of 3,3’-dimethyl-4,4’-dihydroxy azobenzene

4-Amino-*o*-cresol (2.03 g, 13 mmol) was dissolved in 20 mL HCl (2.4N) and stirred under -6°C, while *o*-cresol (2.18 g, 20 mmol) was dissolved in 10 mL NaOH (5.8N) and stirred under -6°C. Then NaNO_2_ (1.66 g, 24 mmol) was dissolved in 6 mL of cold water and added dropwise into the solution of 4-amino-*o*-cresol. Then the mixture was added dropwise into the solution of *o*-cresol. The mixture was stirred with 16h and left to achieve room temperature, then diluted HCl was added to make the mixture acidic (tested by pH test paper). The mixture was then extracted by ethyl acetate, organic layer was washed with brine and dried with MgSO_4_, after which the mixture was filtered, and the solvent was removed reduced pressure. The crude product was purified through column chromatography (eluent = *n*-hexane/ethyl acetate) to yield 2.05 g of a brown solid (65%). ^1^H-NMR (400 MHz, DMSO-d_6_, ppm) δ: 10.02 (s, 2H), 7.62 (d, J = 15.6 Hz, 2H), 7.55 (d-d, J_1_ = 8.4, J_2_ = 2.3 Hz, 2H), 6.92 (d, J = 8.6 Hz, 2H), 2.20 (s, 6H). ^13^C-NMR (101 MHz, DMSO-D_6_, ppm) δ: 159.0, 146.1, 125.7, 125.0, 123.3, 115.7, 17.0.

Synthesis of 3,3’-dimethyl-4,4’-didodecyloxy azobenzene (**1**)

3,3’-dimethyl-4,4’-dihydroxy azobenzene (157 mg, 0.7 mmol) was dissolved in dry acetonitrile under nitrogen, then K_2_CO_3_(719 mg, 5.2 mmol), 1-bromododecane (356 mg, 1.4 mmol) and catalytic amount of 18-crown-6 ether were added. The reaction was stirred and reflux for 1 day. The solvent in mixture was removed under reduced pressure and the residue was purified through column chromatography (eluent = n-hexane/ethyl acetate) and recrystallized from ethanol to yield 136 mg of orange crystal (36%). ^1^H-NMR (400 MHz, CDCl_3_, ppm) δ: 7.73 (d-d, J_1_ = 8.7 Hz, J_2_ = 3.6 Hz, 2H), 7.72 (d, J = 1.9 Hz, 2H), 6.90 (d, J = 9.4 Hz, 2H), 4.04 (t, J = 6.5 Hz, 4H), 2.29 (s, 6H), 1.83 (p, J = 6.6 Hz, 4H), 1.46 – 1.53 (m, 4H), 1.27 – 1.41 (m, 32H), 0.86 – 0.90 (m, 6H). ^13^C-NMR (101 MHz, CDCl_3_, ppm) δ: 159.7, 146.8, 127.8, 123.7, 110.9, 68.6, 32.3, 30.0, 30.0, 30.0, 29.7, 29.7, 29.6, 26.5, 23.1, 16.7, 14.5.

## **Recrystallization**

The single crystals were obtained through two recrystallization methods.

1. Recrystallize from chloroform/ethanol mix solvent. Dissolved 10 mg of **1** in a vial in mixture of 1 mL ethanol and 1 mL chloroform which was sonicated for 5 min to get a clear yellow solution. The cap of vial was loosened, and the solution was left to evaporate at room temperature in dark to grow crystals.
2. Recrystallize from molten state. A sample on a glass was heated on a hot stage under microscope observation. The sample was heated until only one piece of crystal left, then the sample was slowly cooled to grow the crystal.

For single-crystal X-ray diffraction (SXRD) analysis, crystals by melt crystallization are used. In other cases, crystals recrystallized from chloroform/ethanol mix solvent are used.

## **Characterizations**

Nuclear magnetic resonance (NMR) spectra were measured by a Bruker AVANCE 400. Differential scanning calorimetry (DSC) was measured by SII EXSTAR6000. For UV–vis absorption spectroscopy, a JASCO V-780 spectrophotometer was used. Photoirradiation experiments in UV–vis absorption measurement and photothermal effect measurement were performed by LED’s (102LU-4A UV light source with a NS LIGHTNING Co. NULED-102CT AC adaptor for 365 nm, CCS Inc. HLV2-22BL-3W for 465 nm). Light intensity was controlled by a CCS Inc. PJ-1505-2CA analog control unit. An infrared (IR) camera (FLIR i3, Teledyne FLIR LLC) was used to measure the temperature of the crystal sample for photothermal effect measurement, and the temperature control of samples was performed by a TOKAI HIT ThermoPlate. To observe the crystal behavior, optical and polarizing optical photomicrographs were taken by OLYMPUS BX51 microscope equipped with OLYMPUS DP27 digital camera and a HB610 glass filter. Other photoirradiation experiments under microscope were performed by an OLYMPUS U-RFL-T high pressure Hg lamp and optical filters to get in-situ observation on photoirradiation. Light intensity was measured by a Newport 1917-R optical power meter with a 818-ST2-UV/DB photodetector. The three-dimensional images of the crystals were obtained using a KEYENCE VK-X150 laser microscope with a laser wavelength of 658 nm outside the range where the azobenzene samples absorb, and the temperature control of samples was performed by a TOKAI HIT ThermoPlate. In stretching experiments under POM, temperature control of samples was performed by a Linkam10033L heating/cooling stage with a LNP95 cooling system and a T96-PE system controller. In photo-induced crystal stretching experiments under POM, the light intensity was tuned by combination of two Olympus natural density (ND) filters U-25ND6 and U-25ND25. Solid-state absorptions of crystal film were measured using Olympus BX53 with Ocean Optics Flame spectrometer. A Xe lamp was used as a white light source. In all microscopic observation experiments of crystal motion, each crystal was placed on an unmodified cover glass.

## **3D laser scanning microscopy**

To observe and measure the change in size of crystals at different temperatures, a 3D laser microscopy (KEYENCE VK-X150 laser microscope) was employed, which allows for high-resolution measurements of crystal dimensions in three-dimensional space. Temperature was controlled by a TOKAI HIT glass thermal plate.

## **Single-crystal X-ray diffraction (SXRD) analysis**

SXRD experiments for crystals under 170K were performed on a Rigaku XtaLAB mini diffractometer using graphite monochromated Mo-Kα radiation. A sweep of data was done using ω oscillations from -60.0 to 120.0° in 1.0° steps. The exposure rate was 16.0 [sec./°]. Crystal structures were solved by direct methods and refined.

SXRD experiments and analysis for crystals under room temperature and 338K were carried out at BL-8A in Photon Factory (PF), High-Energy Accelerator Research Organization (KEK) with a 2.5-GeV synchrotron radiation source. The wavelength of the X-ray was monochromated to 1.24180 Å with Si(111) double crystals and was calibrated with a CeO_2_ standard sample. All diffraction data were collected using a Rigaku DSC imaging plate system, cell parameter was analyzed by software Rapid AUTO. Using Olex2, the structure was solved with the SHELXT structure solution programs using Intrinsic Phasing. They were then refined with the SHELXL refinement package.^[2–4]^

## **Temperature-dependent powder X-ray diffraction (PXRD) analysis**

PXRD was measured by a Rigaku R-AXIS Ⅳ diffractometer radiation using CuKα radiation (λ = 1.5418 Å, 40 kV, 100 mA). The 2D diffraction patterns were recorded with an imaging plate in a flat camera and digitally transformed into 2theta-I plots. Sandwich samples are prepared by packing crystal powders between two Kapton polyimide films (15 μm thickness) and were placed in a Mettler FP82HT hot stage sample holder to control the temperature. The sample-to-camera length was set to 150.0 mm.

## **Statistical analysis**

The data on temperature-dependent size change of the crystals in different directions in Figure 2a were obtained from three individual crystals, and the average values were calculated.

# **Other supporting figures and tables**

## **DSC data**


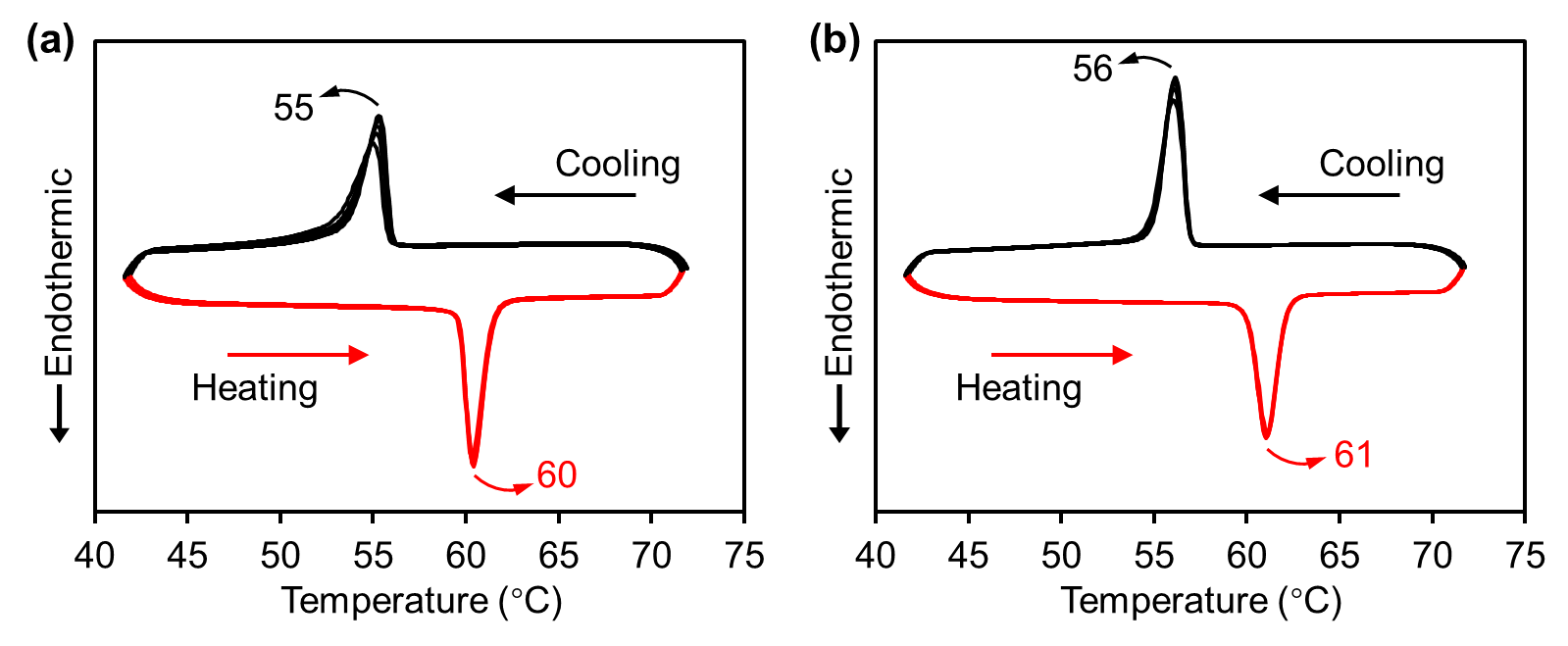


**Figure S1.** Foure and a half cycles of DSC curves of crystal of **1** recrystallized by different methods between 40°C and 75°C with a scanning rate of 5°C/min. DSC curves of (a) crystal by melt crystallization and (b) crystal recrystallized from mix solution of ethanol and chloroform.

## **3D scanning of crystal size analysis**


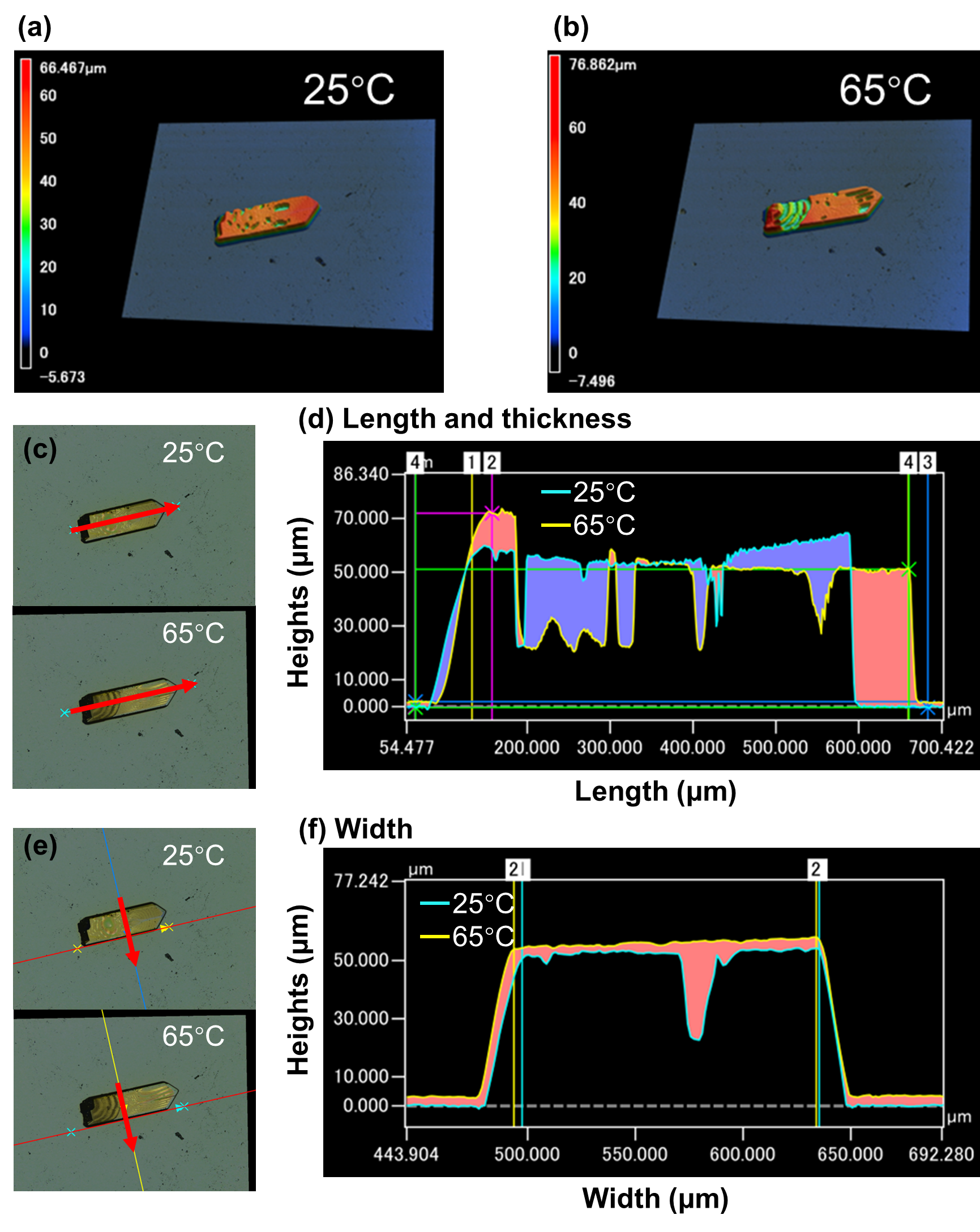


**Figure S2.** 3D images of a crystal at (a) 25°C and (b) 30°C by laser scanning microscope with color mapped based on heights. (c) 3D images of a crystal at 25°C and 30°C with heights measured along the crystal’s long axis. (d) Diagrams of the measured length and thickness (heights) of the crystal. (e) 3D images of a crystal at 25°C and 30°C with width measured. (f) Diagrams of the heights along the width direction of the crystal. Total three different crystals are scanned and measured.

## **Stretching ratio comparison**


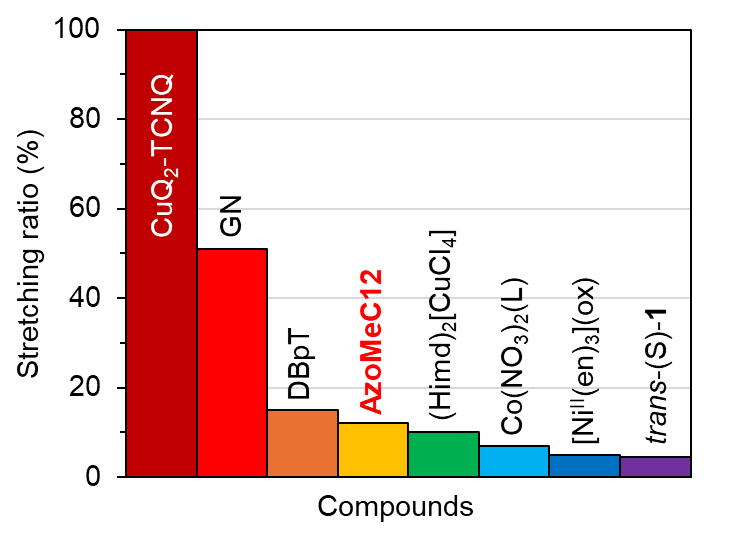


**Figure S3**. Comparison of stretching ratio of AzoMeC12 to other organic crystals with thermal phase transition. CuQ_2_-TCNQ,^[5]^ GN,^[6]^ DBpT,^[7]^ (Himd)_2_[CuCl_4_],^[8]^ Co(NO_3_)_2_(L),^[9]^ [Ni^II^(en)_3_](ox),^[10]^ *trans*-(S)-**1**.^[11]^

## **Heating/cooling rate effect on crystal stretching/shrinking speed**


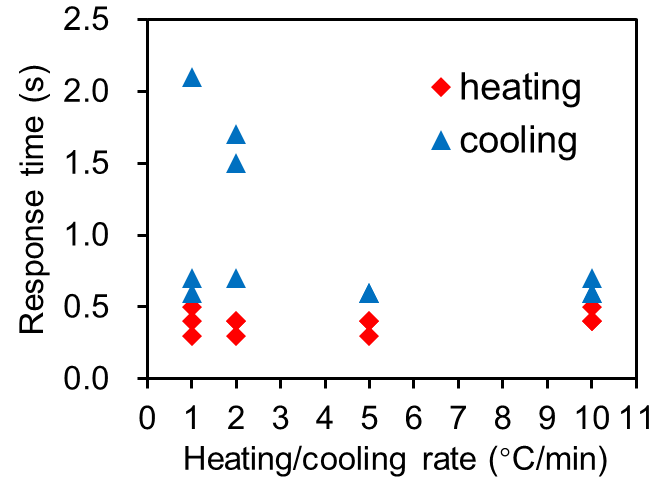


**Figure S4**. The response time for a whole stretching or shrinking process of a single crystal during heating and cooling at various heating/cooling rates (1, 2, 5, 10°C /min). For every heating/cooling rate, the response time is measured three times. The response time is defined as the duration between the onset and completion of the stretching or shrinking process.

## **Crystal structures and intermolecular interactions**

**Table S1.** Summary of X-ray crystallographic data for the single molecular crystal of AzoMeC12 at temperatures of 170 K, 298 K^[a]^, and 338 K.

| Empirical formula | C38H62N2O2 | C38H62N2O2 | C38H62N2O2 |
| --- | --- | --- | --- |
| Temperature/K | 170K | 298K | 338K |
| Formula weight | 578.89 | / | 578.89 |
| Crystal system | Monoclinic | Monoclinic | Monoclinic |
| Space group | *P* 2_1_/*c* | / | *P*2_1_/*c* |
| *a*/Å | 24.80 (2) | 25.1465 (21) | 21.814(6) |
| *b*/Å | 9.425 (7) | 9.5118 (10) | 9.582(3) |
| *c*/Å | 24.48 (2) | 25.2534 (25) | 9.299(2) |
| α/° | 90 | 90 | 90 |
| β/° | 108.746 (11) | 111.2891 (30) | 92.947(4) |
| γ/° | 90 | 90 | 90 |
| V/Å^3^ | 5418 (7) | 5628.1 (9) | 1941.1(8) |
| Z | 6 | / | 2 |
| GOF on F^2^ | 1.046 | / | 0.741 |
| *R*_1_ (*I* > 2σ(*I*)) | 0.1298 | / | 0.0638 |
| *wR*_2_ (all data) | 0.3271 | / | 0.2387 |

[a] Three different diffraction reference frames were used in the analysis of cell parameters of the single crystal at 298K. A comparison of the cell parameters at 298 K and 170 K indicates that the crystal structure remains unchanged at both low and room temperatures.


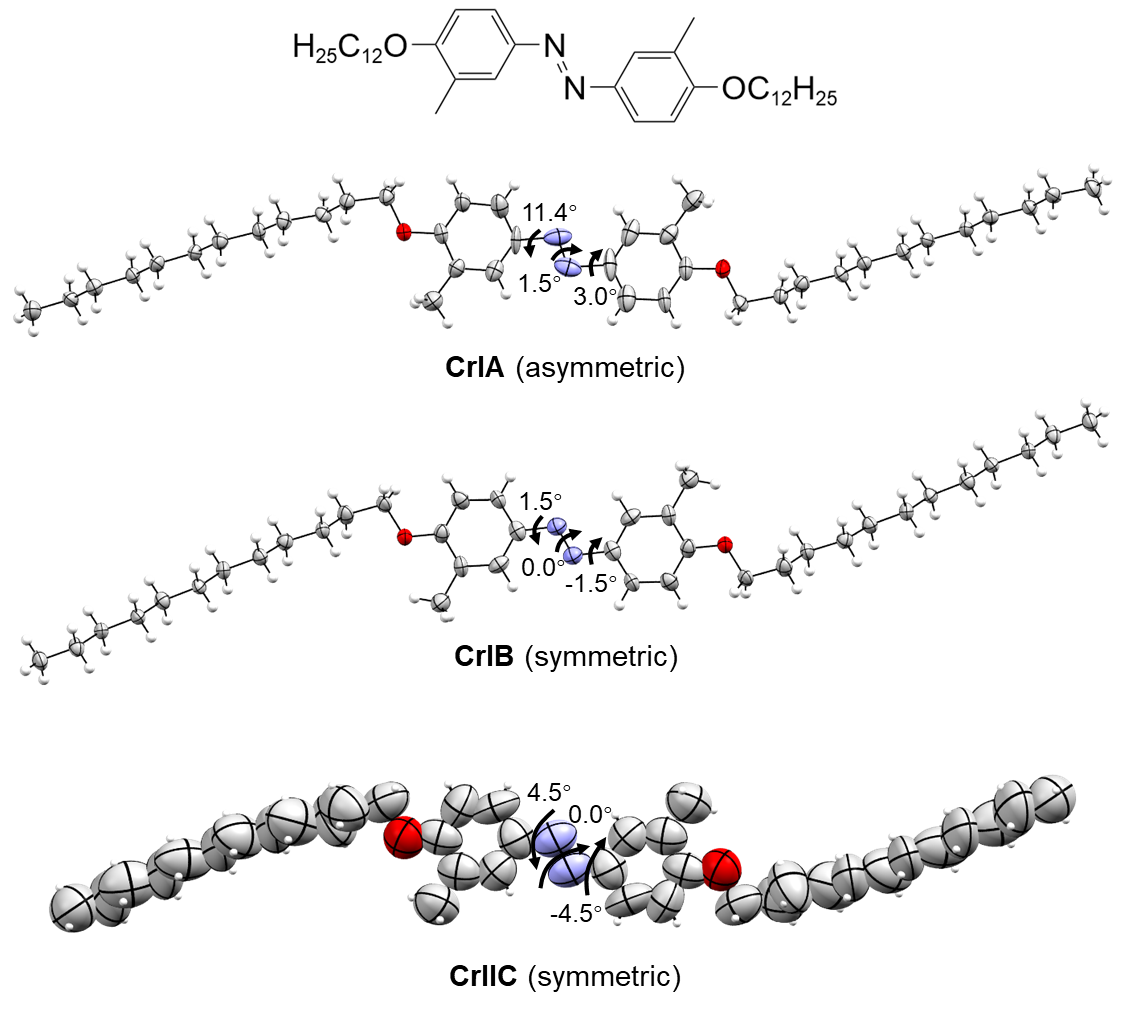


**Figure S5**. Molecular structure of three conformations found in CrI and CrII phases in crystal of AzoMeC12. Thermal ellipsoids are drawn at 50% probability level. Hydrogen atoms are shown as fixed-size spheres of radius 0.2 Å (hydrogen in white, carbon in grey, nitrogen in blue, oxygen in red). The dihedral angles of the azo moieties are shown.


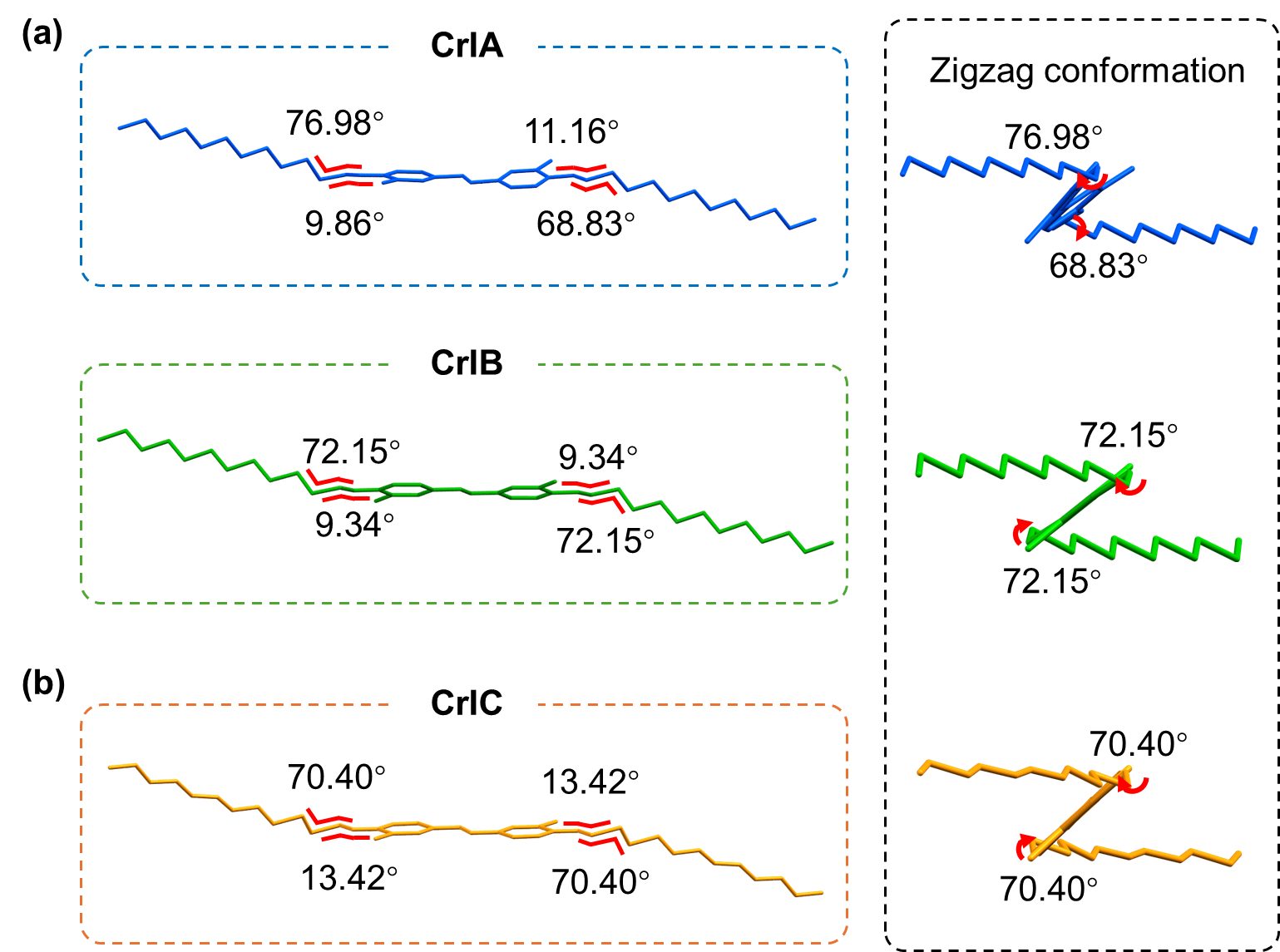


**Figure S6.** Molecular structures of conformers in (a) CrI and (b) CrII. The main dihedral angles are marked. All three conformers exhibit zigzag conformation. Hydrogen atoms have been omitted for clarity.

**
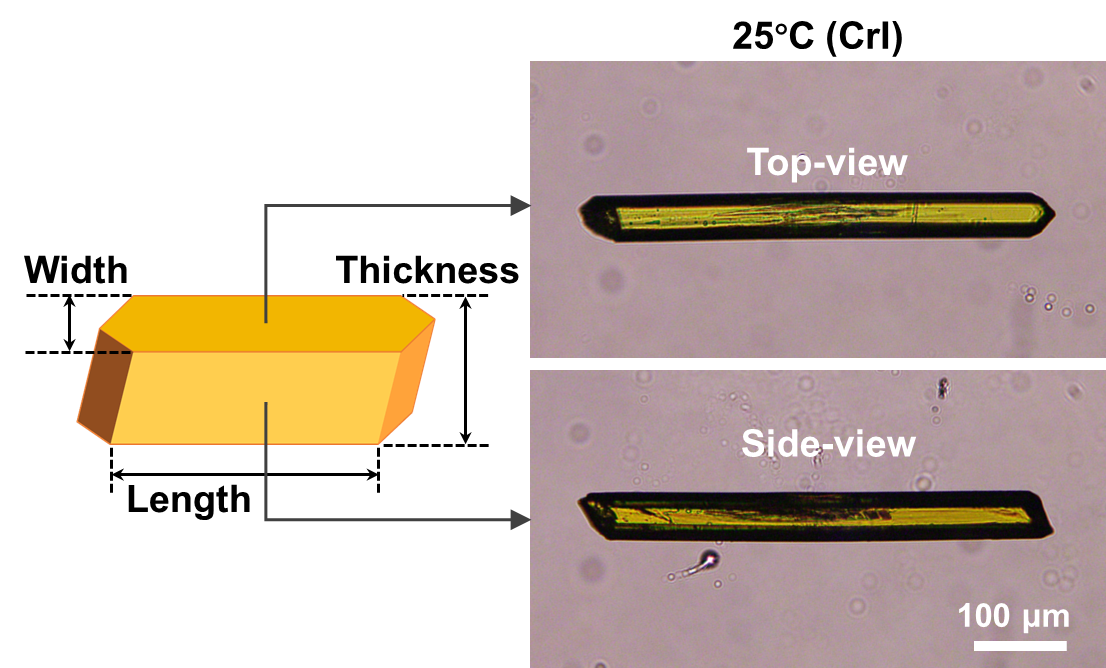
**

**Figure S7**. Illustration of the crystal shape and crystal size along the thickness, length, and width direction.

**
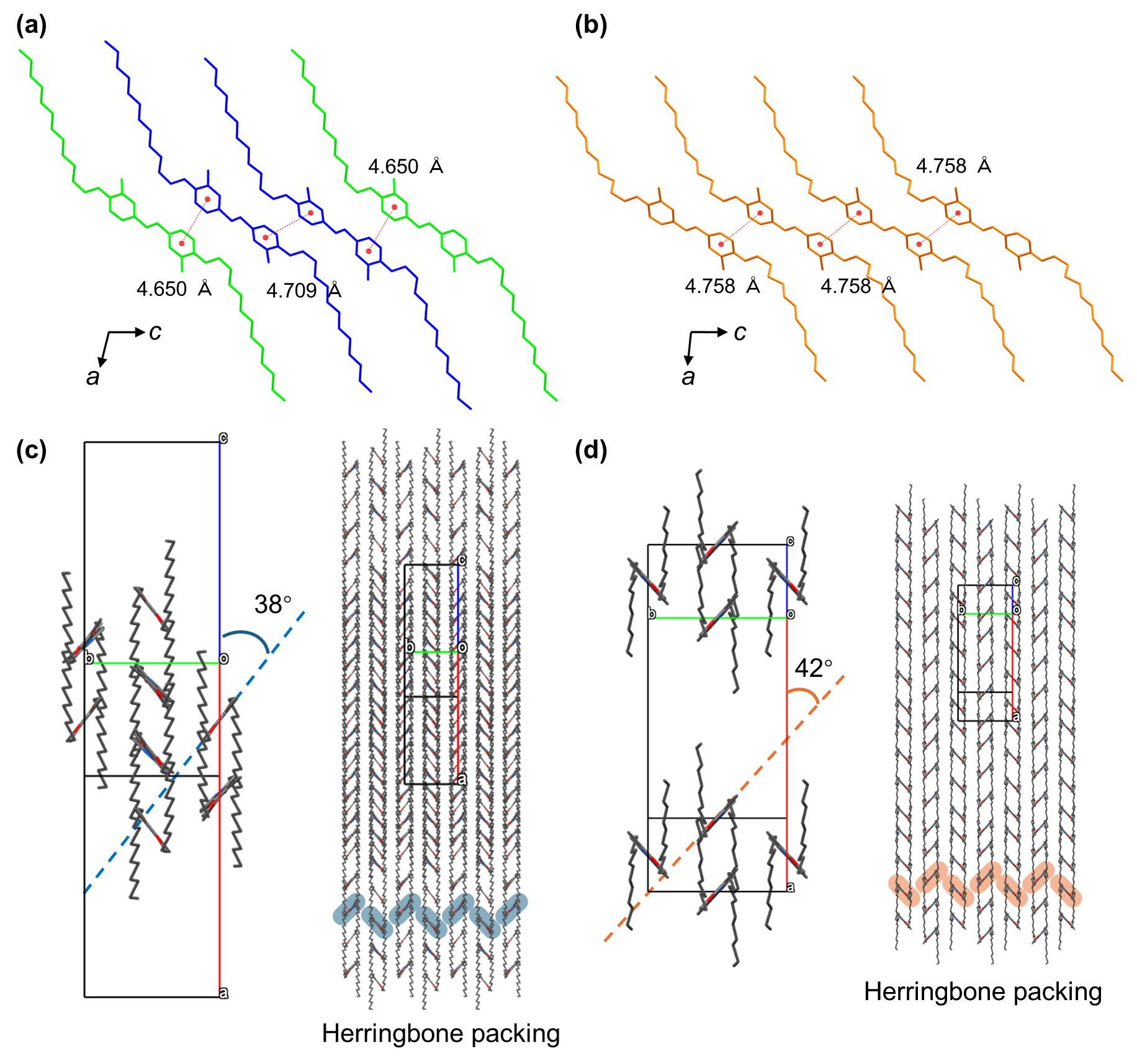
**

**Figure S8.** Distance between parallel-displaced benzene rings that forming π–π stacking columns along *c*-axis in (a) CrI (CrIA in blue, CrIB in green) and (b) CrII phases (CrIIC in orange). Herringbone packing structure at (c) CrI and (d) CrII phases. Leaning angles between azo moieties and (010) face are labelled on CrIB and CrIIC molecules. Hydrogen atoms have been omitted for clarity.

**Table S2.** Crystal size change measured by a laser scanning microscope and crystal size change estimated by SXRD data.

**
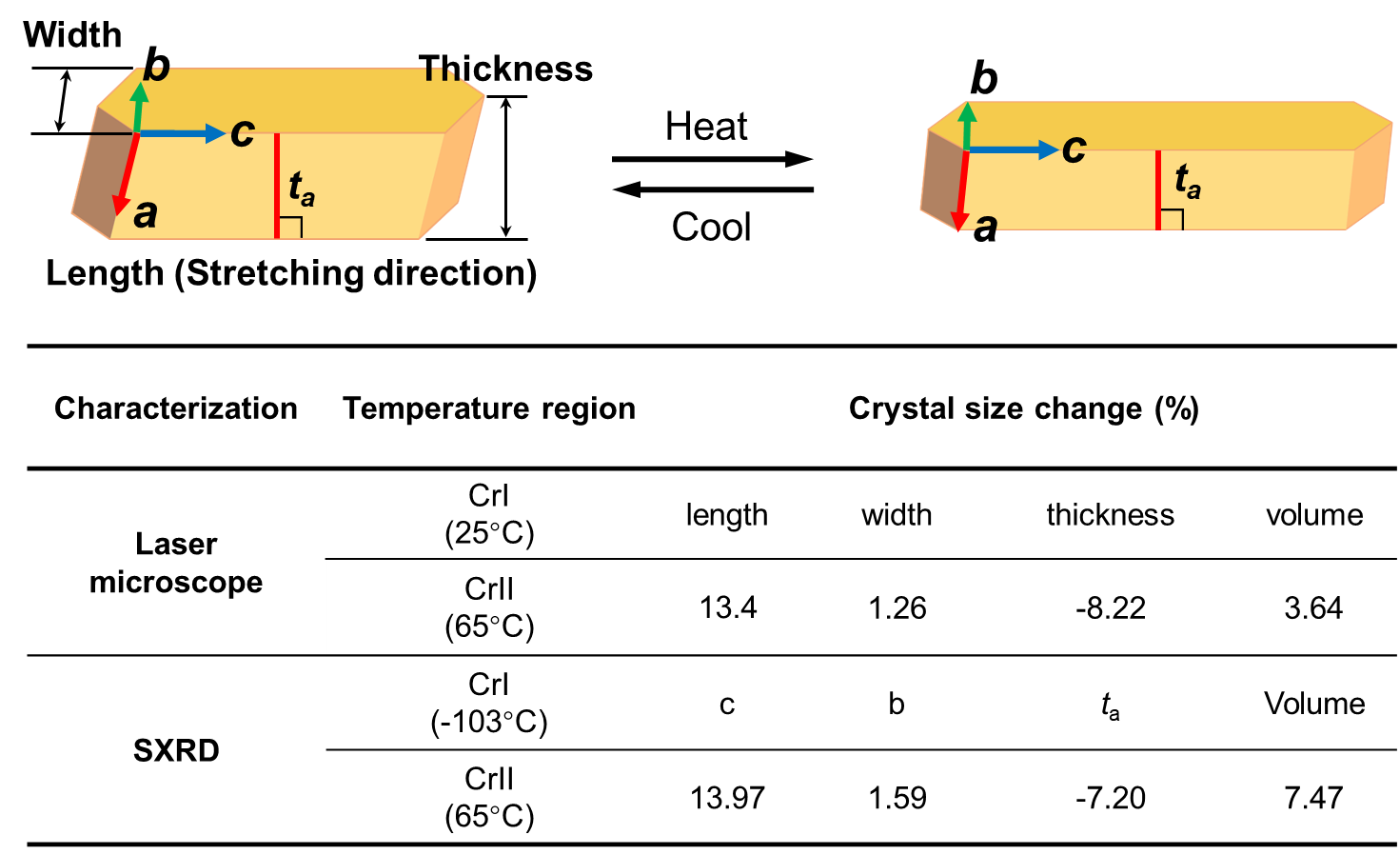
**

| **Characterization** | **Temperature (K)** | **Crystal size change (%)** | | | |
| --- | --- | --- | --- | --- | --- |
| **Laser microscope** | 298K (CrI) | Length | Width | Thickness | Volume |
|  | 338K (CrII) | 13.4 | 1.26 | -8.22 | 3.64 |
| **SXRD** | 298K (CrI) | *c* | *b* | *t_a_* | Volume |
|  | 338K (CrII) | 10.47 | 0.74 | -7.02 | 7.47 |


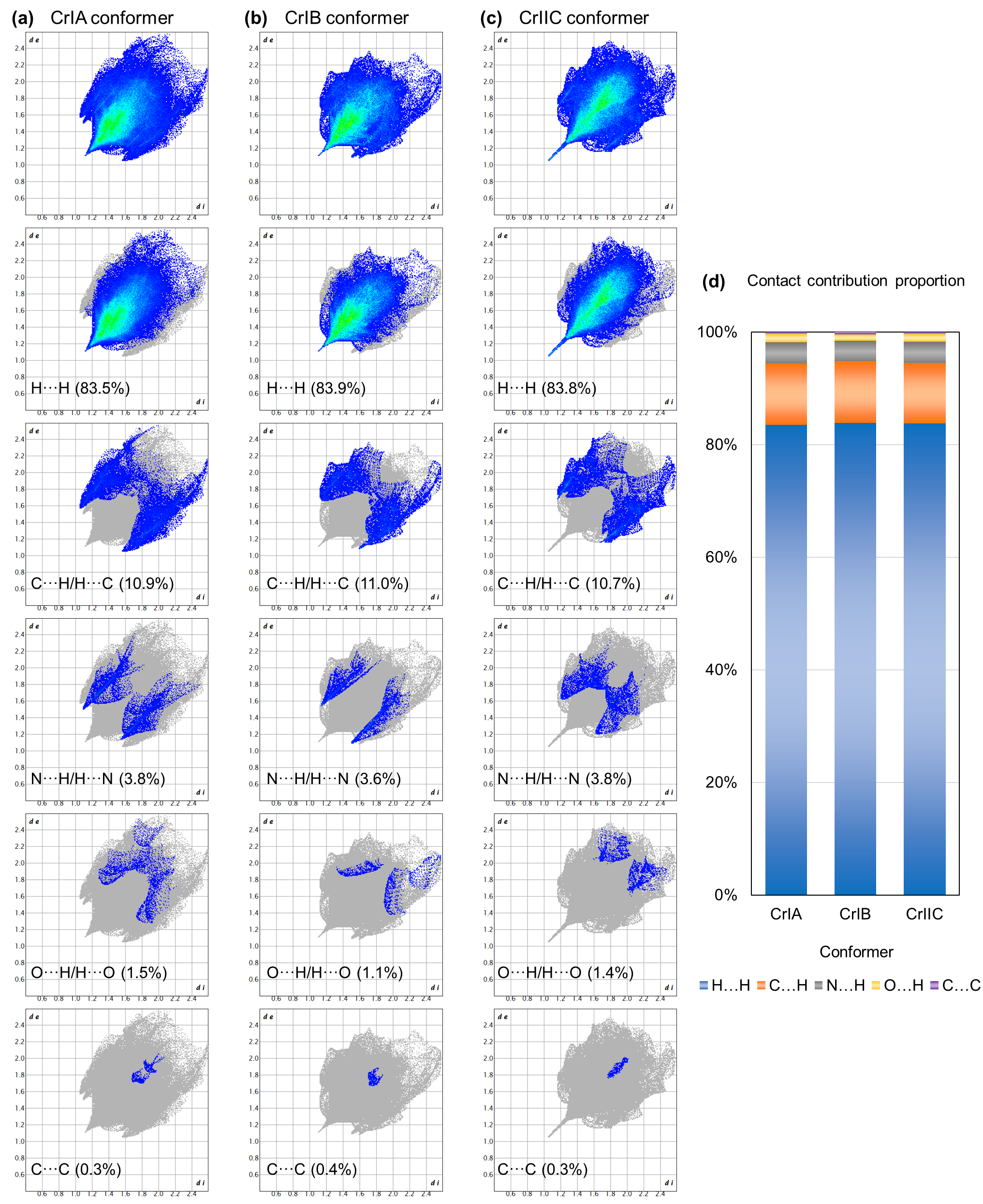


**Figure S9**. Hirshfeld surface 2D fingerprint plots for the distinct intermolecular interactions in (a) CrIA, (b) CrIB, and (c) CrIIC conformers in crystal of AzoMeC12. (d) Contribution proportion of distinct intermolecular interactions in CrIA, CrIB, and CrIIC.


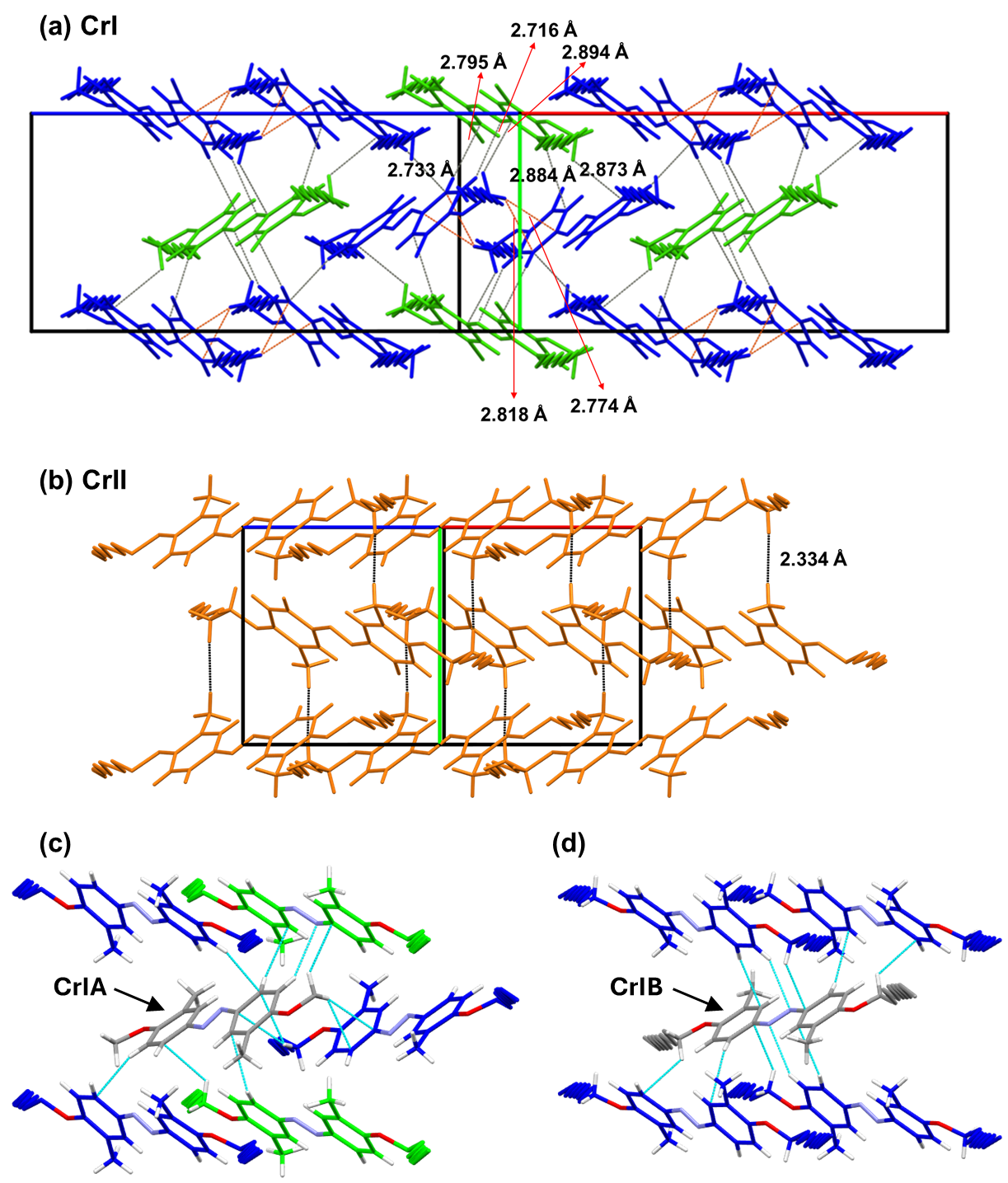


**Figure S10.** Distance between intermolecular short contacts within (a) CrI and (b) CrII phases. Short contacts of a single molecule (colored by element) and connecting molecules (CrIA in blue and CrIB in green) of (c) CrIA and (d) CrIB molecules.

## **Simulated and measured PXRD**


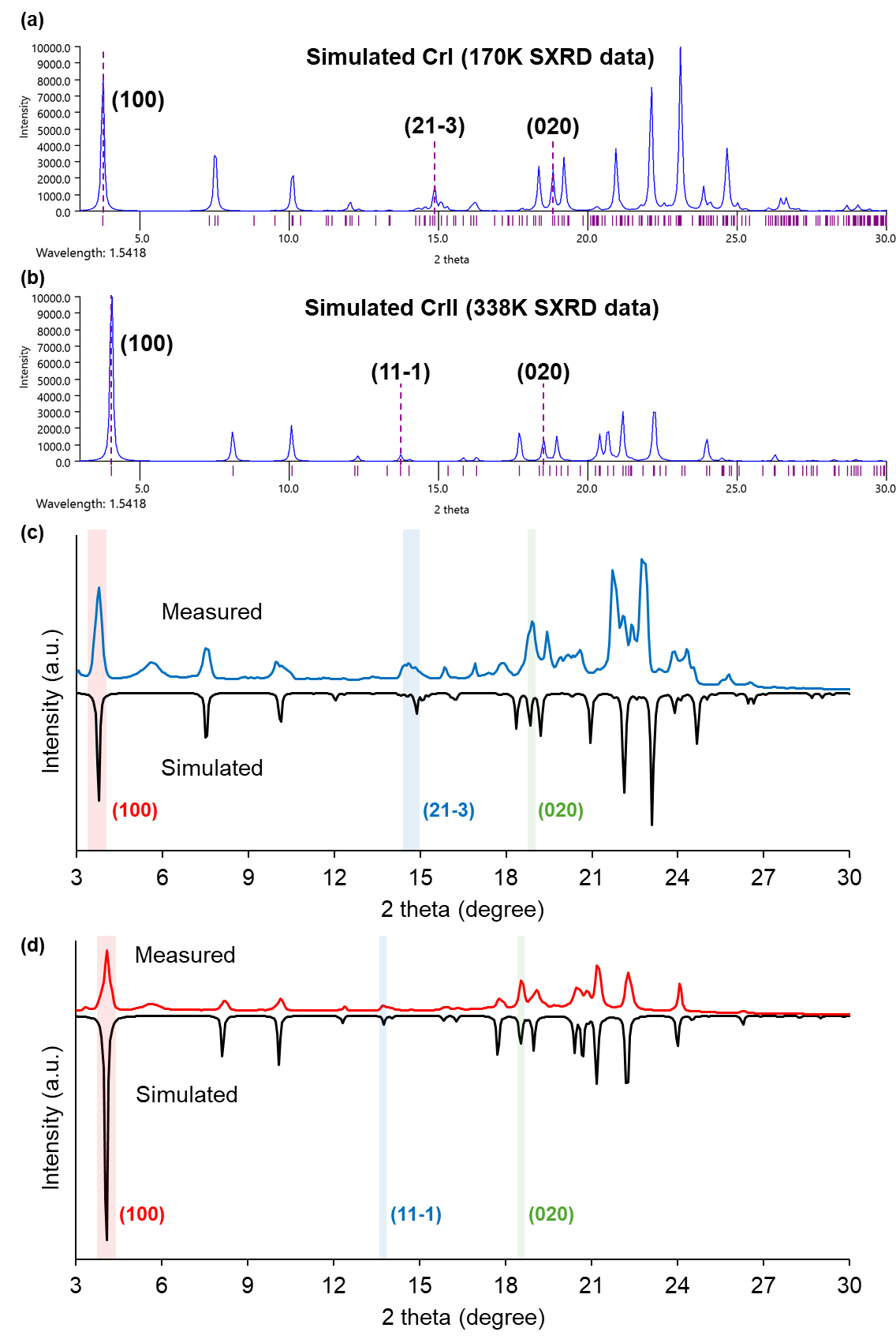


**Figure S11**. Simulated PXRD patterns in (a) CrI and (b) CrII phase from SXRD data. Three representative peaks were chosen considering less overlapping of multiple peaks to avoid misleading of the data process. (c) Comparison of measured PXRD pattern (298K) and PXRD pattern simulated by SXRD data (170K) in CrI phase. (d) Comparison of measured PXRD pattern (338K) and PXRD pattern simulated by SXRD data (338K) in CrII phase.


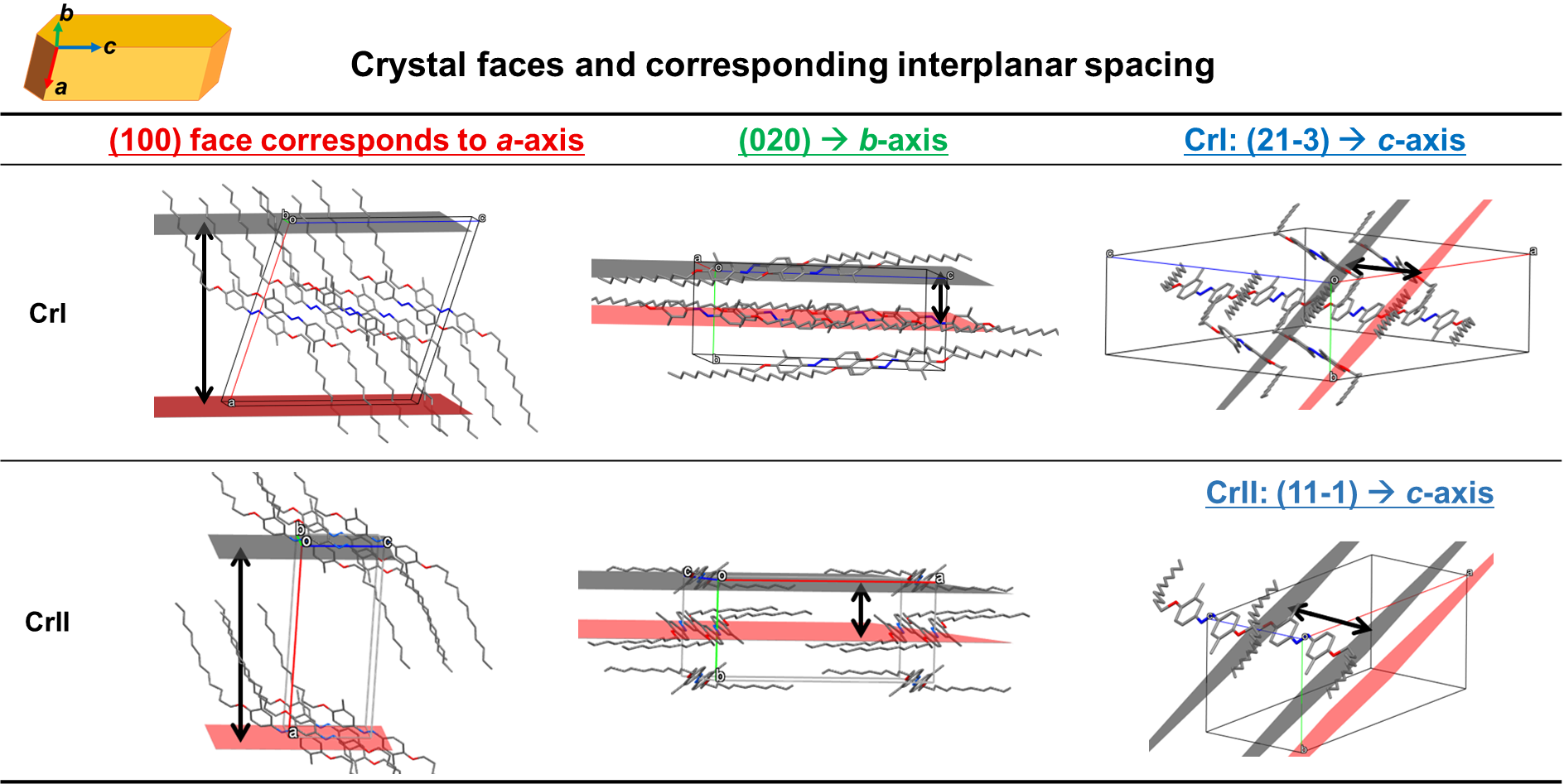


**Figure S12**. Representative crystal faces and their corresponding interplanar spacing.

## **Photothermal effect**


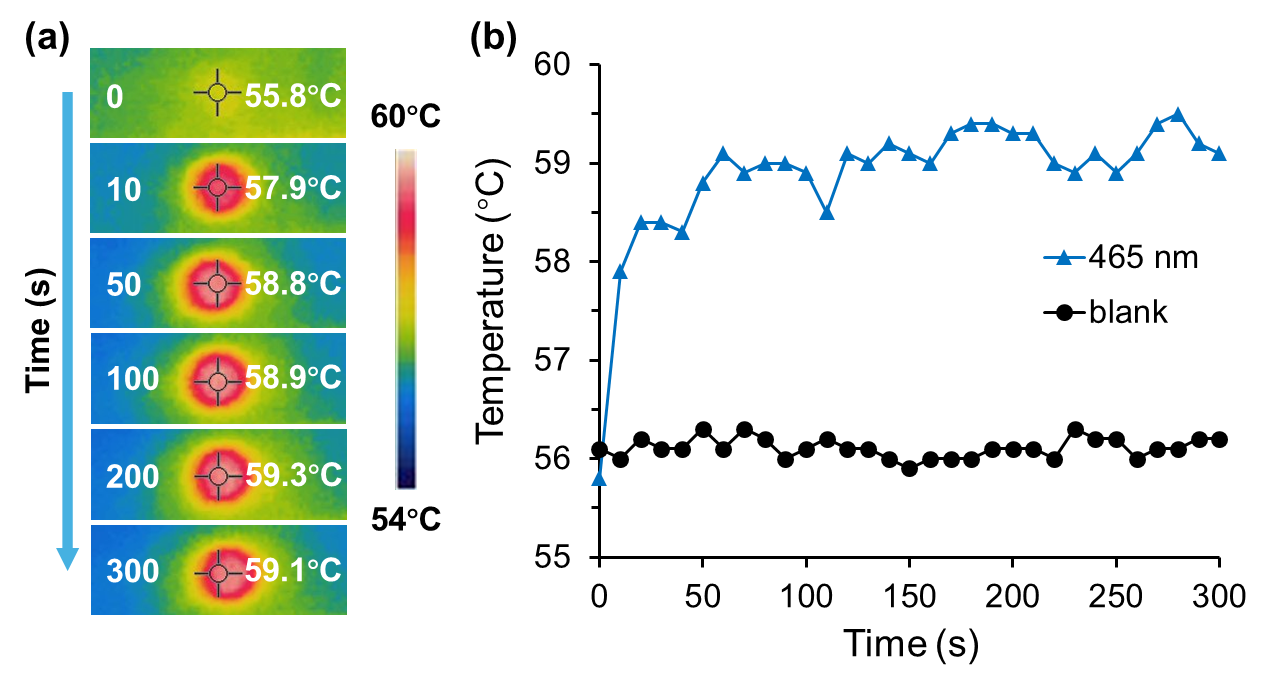


**Figure S13**. (a) IR images of the AzoMeC12 crystalline sample on a cover glass on a heating plate by IR camera at different blue light irradiation times. The irradiated area was approximately 4 mm in diameter. (b) Temperature change of the AzoMeC12 crystalline sample and blank cover glass during continuous irradiation of blue light (465 nm, 0.45W/cm^2^).

## **Photothermal control of crystal stretching/shrinking**


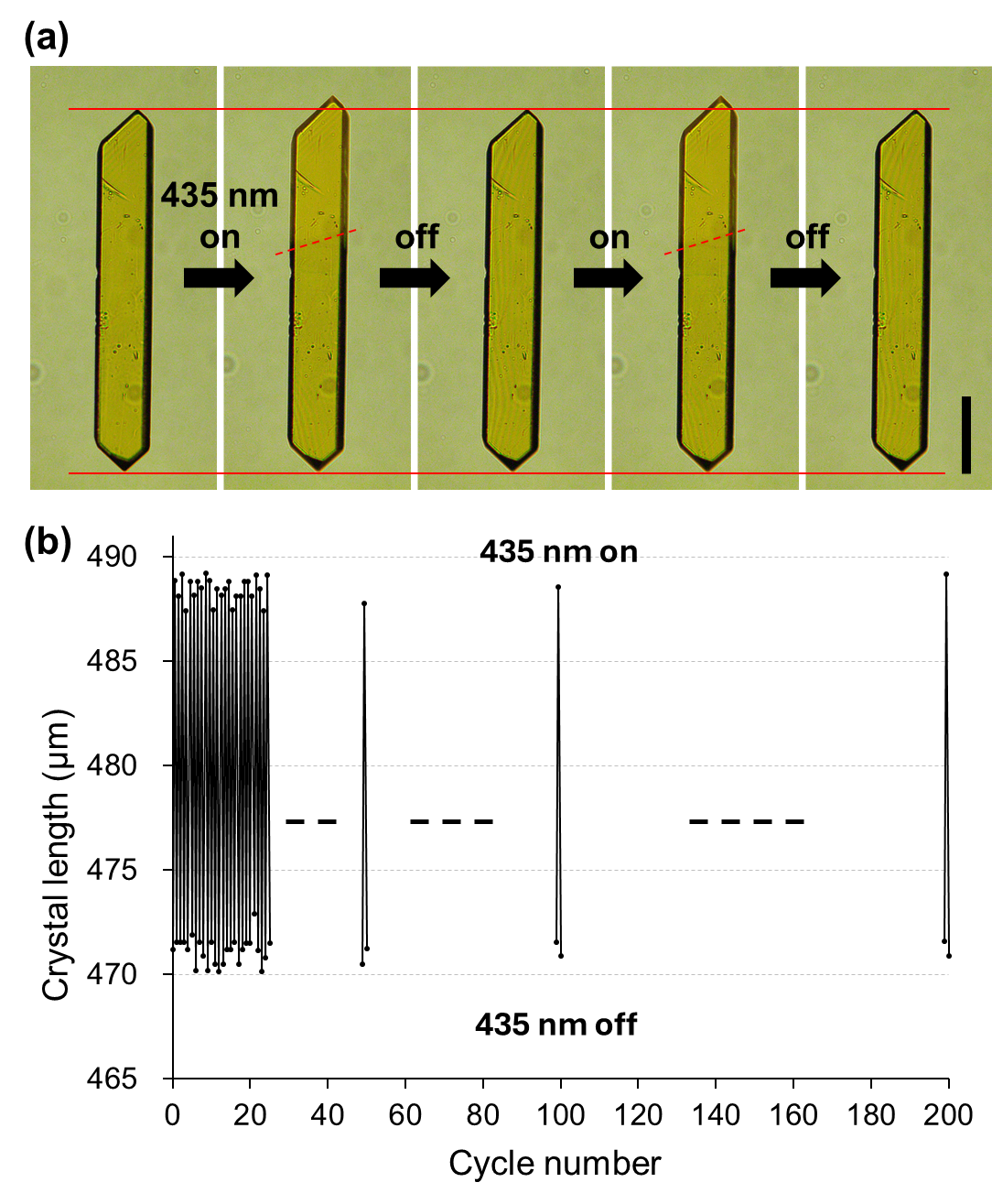


**Figure S14**. (a) Photographs of the local photocontrol of stretching/shrinking of a crystal under 56°C (435 nm light intensity: 3.6 W/cm^2^). Only the upper end of the crystal was irradiated (marked with dash line). Solid lines show the size change of crystal. Scale bar represents 100 μm. (b) Crystal length change under continuous irradiation for 200 cycles measured from (a).

## **UV–vis absorption spectra**

**
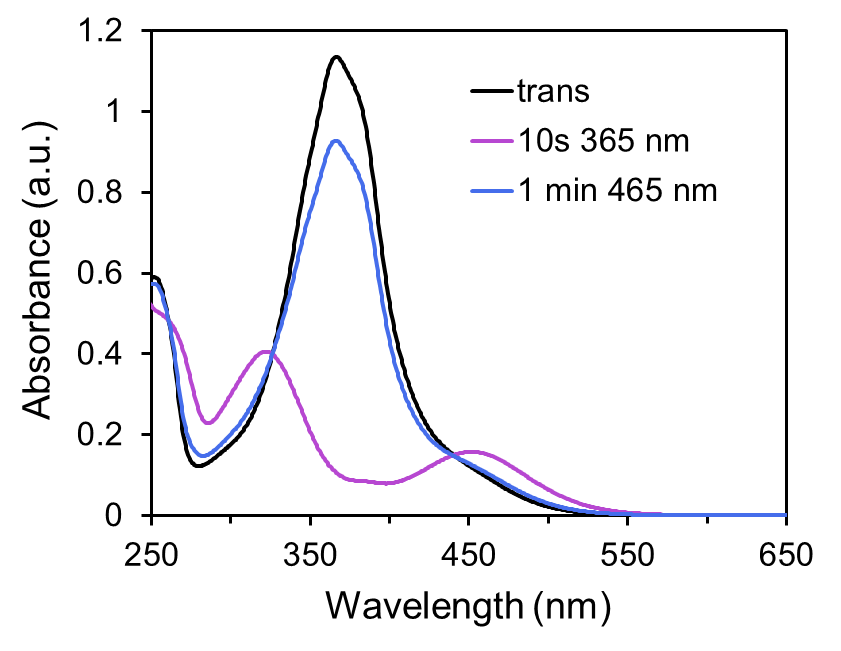
**

**Figure S15.** Absorption spectra of solution of AzoMeC12 (chloroform). Solution kept in dark for one day was used to obtain absorption in original trans state. Then 10 s of 365 nm UV irradiation was employed to reach the photostationary state (cis-rich state), and then 1 min 465 nm blue light irradiation resulted in another photostationary state (trans-rich state).

## **Solid-state absorption spectra**


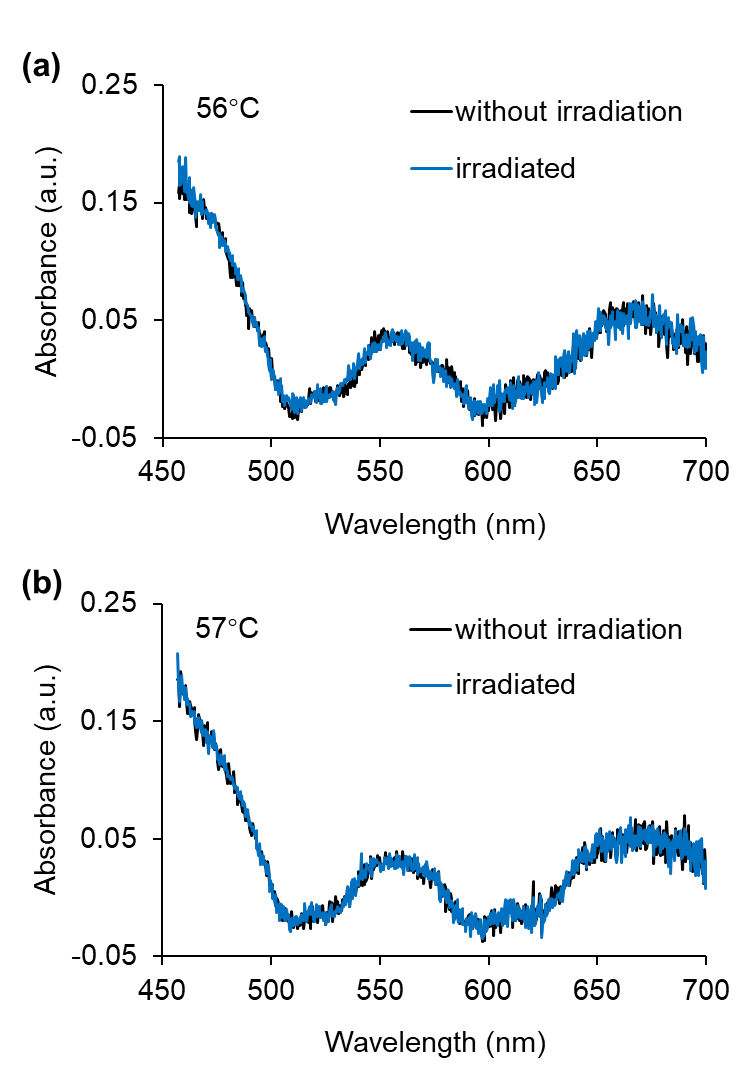


**Figure S16**. Solid-state absorption spectra of a film sample of crystal without (black solid line) and with (blue solid line) 435 nm blue light irradiation (light intensity: 4.8 W/cm^2^) under (a) 56°C and (b) 57°C.

## **Effects of light intensity and temperature on crystal stretching/shrinking**


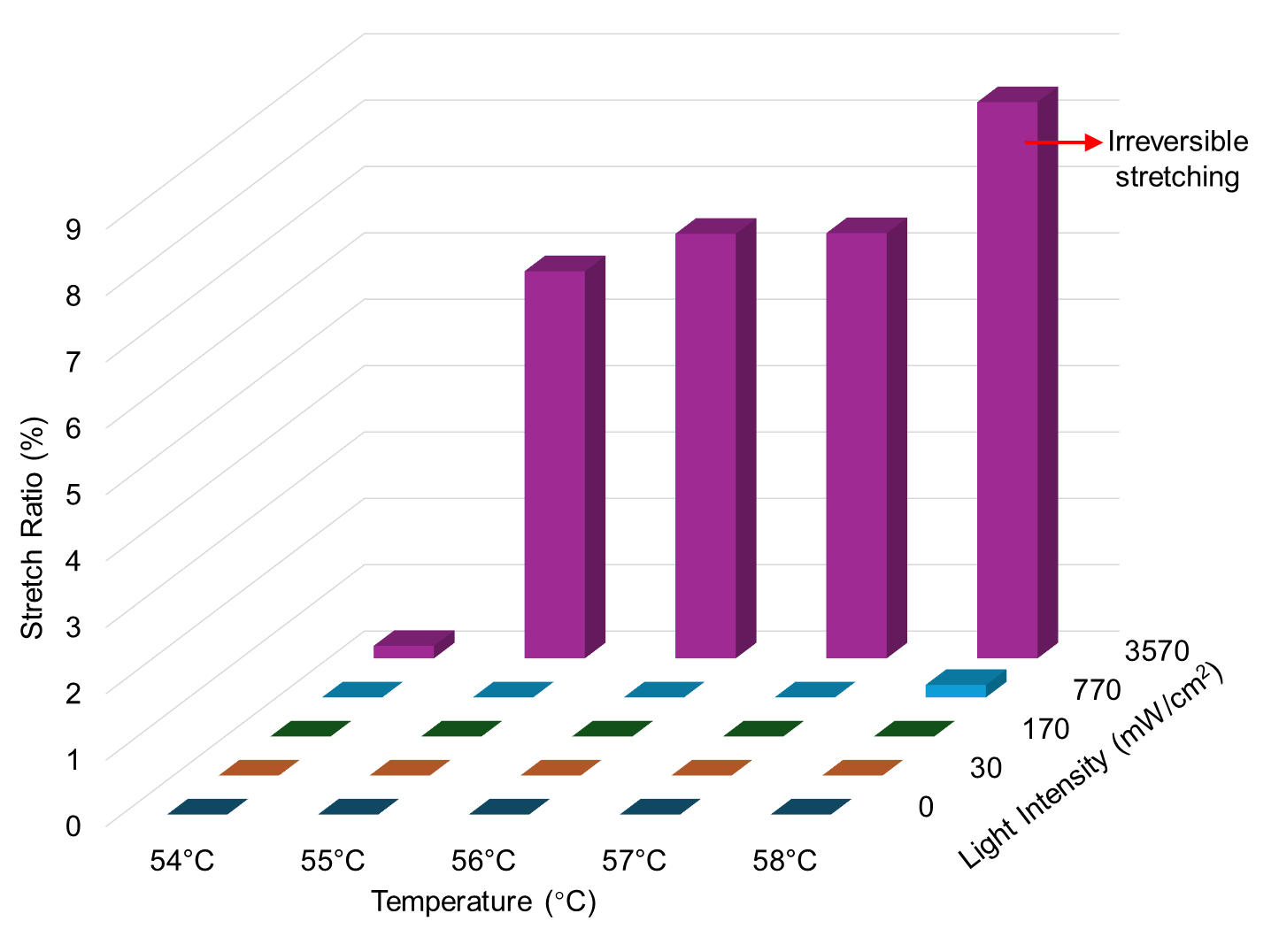


**Figure S17**. Stretch ratio of a crystal under different temperatures and 435 nm light irradiation intensities. The crystal was partially irradiated (approximately 40% of the crystal length from one end) and the stretching ratio was calculated from the change in the total crystal length. The irreversible stretching indicates a complete phase transition under irradiation.

Supplementary Movie 1

Stretching and shrinking motion of a single crystal under heating and cooling observed by microscope at real time.

Supplementary Movie 2

Photo-induced reversible and localized stretching/shrinking of a single crystal observed by microscope at real time (435 nm, light intensity: 3.6 W/cm^2^).

Supplementary Movie 3

Photo-induced reversible and localized stretching/shrinking of a single crystal over 200 cycles observed by microscope and shown at 30× real-time speed (435 nm, light intensity: 3.6 W/cm^2^).

Supplementary Movie 4

A light irradiation on two distinct crystals triggers the abrupt crystal’s stretching of irradiated region, which propels the silica gel microparticle away (435 nm, light intensity: 3.6 W/cm^2^) in real time.

# **References**

[1] E. Uchida, K. Sakaki, Y. Nakamura, R. Azumi, Y. Hirai, H. Akiyama, M. Yoshida, Y. Norikane, “Control of the Orientation and Photoinduced Phase Transitions of Macrocyclic Azobenzene” *Chem. – Eur. J.* **2013**, *19*, 17391–17397.

[2] M. C. Burla, R. Caliandro, M. Camalli, B. Carrozzini, G. L. Cascarano, L. De Caro, C. Giacovazzo, G. Polidori, D. Siliqi, R. Spagna, “IL MILIONE: a suite of computer programs for crystal structure solution of proteins” *J Appl Cryst* **2007**, *40*, 609–613.

[3] O. V. Dolomanov, L. J. Bourhis, R. J. Gildea, J. A. K. Howard, H. Puschmann, “OLEX2: a complete structure solution, refinement and analysis program” *J Appl Cryst* **2009**, *42*, 339–341.

[4] G. M. Sheldrick, “Crystal structure refinement with SHELXL” *Acta Crystallogr C Struct Chem* **2015**, *71*, 3–8.

[5] G. Liu, J. Liu, Y. Liu, X. Tao, “Oriented Single-Crystal-to-Single-Crystal Phase Transition with Dramatic Changes in the Dimensions of Crystals” *J. Am. Chem. Soc.* **2014**, *136*, 590–593.

[6] D. P. Karothu, R. Ferreira, G. Dushaq, E. Ahmed, L. Catalano, J. M. Halabi, Z. Alhaddad, I. Tahir, L. Li, S. Mohamed, M. Rasras, P. Naumov, “Exceptionally high work density of a ferroelectric dynamic organic crystal around room temperature” *Nat. Commun.* **2022**, *13*, 2823.

[7] Z. Wang, R. Shi, I. Tahir, D. P. Karothu, P. Cheng, W. Han, L. Li, Y. Zheng, P. Naumov, J. Xu, X.-H. Bu, “Thiophene Sulfone Single Crystal as a Reversible Thermoelastic Linear Actuator with an Extended Stroke and Second-Harmonic Generation Switching” *J. Am. Chem. Soc.* **2025**, *147*, 7749–7756.

[8] Z.-S. Yao, H. Guan, Y. Shiota, C.-T. He, X.-L. Wang, S.-Q. Wu, X. Zheng, S.-Q. Su, K. Yoshizawa, X. Kong, O. Sato, J. Tao, “Giant anisotropic thermal expansion actuated by thermodynamically assisted reorientation of imidazoliums in a single crystal” *Nat. Commun.* **2019**, *10*, 4805.

[9] S.-Q. Su, T. Kamachi, Z.-S. Yao, Y.-G. Huang, Y. Shiota, K. Yoshizawa, N. Azuma, Y. Miyazaki, M. Nakano, G. Maruta, S. Takeda, S. Kang, S. Kanegawa, O. Sato, “Assembling an alkyl rotor to access abrupt and reversible crystalline deformation of a cobalt(II) complex” *Nat. Commun.* **2015**, *6*, 8810.

[10] Z.-S. Yao, M. Mito, T. Kamachi, Y. Shiota, K. Yoshizawa, N. Azuma, Y. Miyazaki, K. Takahashi, K. Zhang, T. Nakanishi, S. Kang, S. Kanegawa, O. Sato, “Molecular motor-driven abrupt anisotropic shape change in a single crystal of a Ni complex” *Nat. Chem.* **2014**, *6*, 1079–1083.

[11] T. Taniguchi, H. Sugiyama, H. Uekusa, M. Shiro, T. Asahi, H. Koshima, “Walking and rolling of crystals induced thermally by phase transition” *Nat. Commun.* **2018**, *9*, 538.
